# The database of the PREDICTS (Projecting Responses of Ecological Diversity In Changing Terrestrial Systems) project

**DOI:** 10.1002/ece3.2579

**Published:** 2016-12-16

**Authors:** Lawrence N. Hudson, Tim Newbold, Sara Contu, Samantha L. L. Hill, Igor Lysenko, Adriana De Palma, Helen R. P. Phillips, Tamera I. Alhusseini, Felicity E. Bedford, Dominic J. Bennett, Hollie Booth, Victoria J. Burton, Charlotte W. T. Chng, Argyrios Choimes, David L. P. Correia, Julie Day, Susy Echeverría‐Londoño, Susan R. Emerson, Di Gao, Morgan Garon, Michelle L. K. Harrison, Daniel J. Ingram, Martin Jung, Victoria Kemp, Lucinda Kirkpatrick, Callum D. Martin, Yuan Pan, Gwilym D. Pask‐Hale, Edwin L. Pynegar, Alexandra N. Robinson, Katia Sanchez‐Ortiz, Rebecca A. Senior, Benno I. Simmons, Hannah J. White, Hanbin Zhang, Job Aben, Stefan Abrahamczyk, Gilbert B. Adum, Virginia Aguilar‐Barquero, Marcelo A. Aizen, Belén Albertos, E. L. Alcala, Maria del Mar Alguacil, Audrey Alignier, Marc Ancrenaz, Alan N. Andersen, Enrique Arbeláez‐Cortés, Inge Armbrecht, Víctor Arroyo‐Rodríguez, Tom Aumann, Jan C. Axmacher, Badrul Azhar, Adrián B. Azpiroz, Lander Baeten, Adama Bakayoko, András Báldi, John E. Banks, Sharad K. Baral, Jos Barlow, Barbara I. P. Barratt, Lurdes Barrico, Paola Bartolommei, Diane M. Barton, Yves Basset, Péter Batáry, Adam J. Bates, Bruno Baur, Erin M. Bayne, Pedro Beja, Suzan Benedick, Åke Berg, Henry Bernard, Nicholas J. Berry, Dinesh Bhatt, Jake E. Bicknell, Jochen H. Bihn, Robin J. Blake, Kadiri S. Bobo, Roberto Bóçon, Teun Boekhout, Katrin Böhning‐Gaese, Kevin J. Bonham, Paulo A. V. Borges, Sérgio H. Borges, Céline Boutin, Jérémy Bouyer, Cibele Bragagnolo, Jodi S. Brandt, Francis Q. Brearley, Isabel Brito, Vicenç Bros, Jörg Brunet, Grzegorz Buczkowski, Christopher M. Buddle, Rob Bugter, Erika Buscardo, Jörn Buse, Jimmy Cabra‐García, Nilton C. Cáceres, Nicolette L. Cagle, María Calviño‐Cancela, Sydney A. Cameron, Eliana M. Cancello, Rut Caparrós, Pedro Cardoso, Dan Carpenter, Tiago F. Carrijo, Anelena L. Carvalho, Camila R. Cassano, Helena Castro, Alejandro A. Castro‐Luna, Cerda B. Rolando, Alexis Cerezo, Kim Alan Chapman, Matthieu Chauvat, Morten Christensen, Francis M. Clarke, Daniel F.R. Cleary, Giorgio Colombo, Stuart P. Connop, Michael D. Craig, Leopoldo Cruz‐López, Saul A. Cunningham, Biagio D'Aniello, Neil D'Cruze, Pedro Giovâni da Silva, Martin Dallimer, Emmanuel Danquah, Ben Darvill, Jens Dauber, Adrian L. V. Davis, Jeff Dawson, Claudio de Sassi, Benoit de Thoisy, Olivier Deheuvels, Alain Dejean, Jean‐Louis Devineau, Tim Diekötter, Jignasu V. Dolia, Erwin Domínguez, Yamileth Dominguez‐Haydar, Silvia Dorn, Isabel Draper, Niels Dreber, Bertrand Dumont, Simon G. Dures, Mats Dynesius, Lars Edenius, Paul Eggleton, Felix Eigenbrod, Zoltán Elek, Martin H. Entling, Karen J. Esler, Ricardo F. de Lima, Aisyah Faruk, Nina Farwig, Tom M. Fayle, Antonio Felicioli, Annika M. Felton, Roderick J. Fensham, Ignacio C. Fernandez, Catarina C. Ferreira, Gentile F. Ficetola, Cristina Fiera, Bruno K. C. Filgueiras, Hüseyin K. Fırıncıoğlu, David Flaspohler, Andreas Floren, Steven J. Fonte, Anne Fournier, Robert E. Fowler, Markus Franzén, Lauchlan H. Fraser, Gabriella M. Fredriksson, Geraldo B. Freire, Tiago L. M. Frizzo, Daisuke Fukuda, Dario Furlani, René Gaigher, Jörg U. Ganzhorn, Karla P. García, Juan C. Garcia‐R, Jenni G. Garden, Ricardo Garilleti, Bao‐Ming Ge, Benoit Gendreau‐Berthiaume, Philippa J. Gerard, Carla Gheler‐Costa, Benjamin Gilbert, Paolo Giordani, Simonetta Giordano, Carly Golodets, Laurens G. L. Gomes, Rachelle K. Gould, Dave Goulson, Aaron D. Gove, Laurent Granjon, Ingo Grass, Claudia L. Gray, James Grogan, Weibin Gu, Moisès Guardiola, Nihara R. Gunawardene, Alvaro G. Gutierrez, Doris L. Gutiérrez‐Lamus, Daniela H. Haarmeyer, Mick E. Hanley, Thor Hanson, Nor R. Hashim, Shombe N. Hassan, Richard G. Hatfield, Joseph E. Hawes, Matt W. Hayward, Christian Hébert, Alvin J. Helden, John‐André Henden, Philipp Henschel, Lionel Hernández, James P. Herrera, Farina Herrmann, Felix Herzog, Diego Higuera‐Diaz, Branko Hilje, Hubert Höfer, Anke Hoffmann, Finbarr G. Horgan, Elisabeth Hornung, Roland Horváth, Kristoffer Hylander, Paola Isaacs‐Cubides, Hiroaki Ishida, Masahiro Ishitani, Carmen T. Jacobs, Víctor J. Jaramillo, Birgit Jauker, F. Jiménez Hernández, McKenzie F. Johnson, Virat Jolli, Mats Jonsell, S. Nur Juliani, Thomas S. Jung, Vena Kapoor, Heike Kappes, Vassiliki Kati, Eric Katovai, Klaus Kellner, Michael Kessler, Kathryn R. Kirby, Andrew M. Kittle, Mairi E. Knight, Eva Knop, Florian Kohler, Matti Koivula, Annette Kolb, Mouhamadou Kone, Ádám Kőrösi, Jochen Krauss, Ajith Kumar, Raman Kumar, David J. Kurz, Alex S. Kutt, Thibault Lachat, Victoria Lantschner, Francisco Lara, Jesse R. Lasky, Steven C. Latta, William F. Laurance, Patrick Lavelle, Violette Le Féon, Gretchen LeBuhn, Jean‐Philippe Légaré, Valérie Lehouck, María V. Lencinas, Pia E. Lentini, Susan G. Letcher, Qi Li, Simon A. Litchwark, Nick A. Littlewood, Yunhui Liu, Nancy Lo‐Man‐Hung, Carlos A. López‐Quintero, Mounir Louhaichi, Gabor L. Lövei, Manuel Esteban Lucas‐Borja, Victor H. Luja, Matthew S. Luskin, M Cristina MacSwiney G, Kaoru Maeto, Tibor Magura, Neil Aldrin Mallari, Louise A. Malone, Patrick K. Malonza, Jagoba Malumbres‐Olarte, Salvador Mandujano, Inger E. Måren, Erika Marin‐Spiotta, Charles J. Marsh, E. J. P. Marshall, Eliana Martínez, Guillermo Martínez Pastur, David Moreno Mateos, Margaret M. Mayfield, Vicente Mazimpaka, Jennifer L. McCarthy, Kyle P. McCarthy, Quinn S. McFrederick, Sean McNamara, Nagore G. Medina, Rafael Medina, Jose L. Mena, Estefania Mico, Grzegorz Mikusinski, Jeffrey C. Milder, James R. Miller, Daniel R. Miranda‐Esquivel, Melinda L. Moir, Carolina L. Morales, Mary N. Muchane, Muchai Muchane, Sonja Mudri‐Stojnic, A. Nur Munira, Antonio Muoñz‐Alonso, B. F. Munyekenye, Robin Naidoo, A. Naithani, Michiko Nakagawa, Akihiro Nakamura, Yoshihiro Nakashima, Shoji Naoe, Guiomar Nates‐Parra, Dario A. Navarrete Gutierrez, Luis Navarro‐Iriarte, Paul K. Ndang'ang'a, Eike L. Neuschulz, Jacqueline T. Ngai, Violaine Nicolas, Sven G. Nilsson, Norbertas Noreika, Olivia Norfolk, Jorge Ari Noriega, David A. Norton, Nicole M. Nöske, A. Justin Nowakowski, Catherine Numa, Niall O'Dea, Patrick J. O'Farrell, William Oduro, Sabine Oertli, Caleb Ofori‐Boateng, Christopher Omamoke Oke, Vicencio Oostra, Lynne M. Osgathorpe, Samuel Eduardo Otavo, Navendu V. Page, Juan Paritsis, Alejandro Parra‐H, Luke Parry, Guy Pe'er, Peter B. Pearman, Nicolás Pelegrin, Raphaël Pélissier, Carlos A. Peres, Pablo L. Peri, Anna S. Persson, Theodora Petanidou, Marcell K. Peters, Rohan S. Pethiyagoda, Ben Phalan, T. Keith Philips, Finn C. Pillsbury, Jimmy Pincheira‐Ulbrich, Eduardo Pineda, Joan Pino, Jaime Pizarro‐Araya, A. J. Plumptre, Santiago L. Poggio, Natalia Politi, Pere Pons, Katja Poveda, Eileen F. Power, Steven J. Presley, Vânia Proença, Marino Quaranta, Carolina Quintero, Romina Rader, B. R. Ramesh, Martha P. Ramirez‐Pinilla, Jai Ranganathan, Claus Rasmussen, Nicola A. Redpath‐Downing, J. Leighton Reid, Yana T. Reis, José M. Rey Benayas, Juan Carlos Rey‐Velasco, Chevonne Reynolds, Danilo Bandini Ribeiro, Miriam H. Richards, Barbara A. Richardson, Michael J. Richardson, Rodrigo Macip Ríos, Richard Robinson, Carolina A. Robles, Jörg Römbke, Luz Piedad Romero‐Duque, Matthias Rös, Loreta Rosselli, Stephen J. Rossiter, Dana S. Roth, T'ai H. Roulston, Laurent Rousseau, André V. Rubio, Jean‐Claude Ruel, Jonathan P. Sadler, Szabolcs Sáfián, Romeo A. Saldaña‐Vázquez, Katerina Sam, Ulrika Samnegård, Joana Santana, Xavier Santos, Jade Savage, Nancy A. Schellhorn, Menno Schilthuizen, Ute Schmiedel, Christine B. Schmitt, Nicole L. Schon, Christof Schüepp, Katharina Schumann, Oliver Schweiger, Dawn M. Scott, Kenneth A. Scott, Jodi L. Sedlock, Steven S. Seefeldt, Ghazala Shahabuddin, Graeme Shannon, Douglas Sheil, Frederick H. Sheldon, Eyal Shochat, Stefan J. Siebert, Fernando A. B. Silva, Javier A. Simonetti, Eleanor M. Slade, Jo Smith, Allan H. Smith‐Pardo, Navjot S. Sodhi, Eduardo J. Somarriba, Ramón A. Sosa, Grimaldo Soto Quiroga, Martin‐Hugues St‐Laurent, Brian M. Starzomski, Constanti Stefanescu, Ingolf Steffan‐Dewenter, Philip C. Stouffer, Jane C. Stout, Ayron M. Strauch, Matthew J. Struebig, Zhimin Su, Marcela Suarez‐Rubio, Shinji Sugiura, Keith S. Summerville, Yik‐Hei Sung, Hari Sutrisno, Jens‐Christian Svenning, Tiit Teder, Caragh G. Threlfall, Anu Tiitsaar, Jacqui H. Todd, Rebecca K. Tonietto, Ignasi Torre, Béla Tóthmérész, Teja Tscharntke, Edgar C. Turner, Jason M. Tylianakis, Marcio Uehara‐Prado, Nicolas Urbina‐Cardona, Denis Vallan, Adam J. Vanbergen, Heraldo L. Vasconcelos, Kiril Vassilev, Hans A. F. Verboven, Maria João Verdasca, José R. Verdú, Carlos H. Vergara, Pablo M. Vergara, Jort Verhulst, Massimiliano Virgilio, Lien Van Vu, Edward M. Waite, Tony R. Walker, Hua‐Feng Wang, Yanping Wang, James I. Watling, Britta Weller, Konstans Wells, Catrin Westphal, Edward D. Wiafe, Christopher D. Williams, Michael R. Willig, John C. Z. Woinarski, Jan H. D. Wolf, Volkmar Wolters, Ben A. Woodcock, Jihua Wu, Joseph M. Wunderle, Yuichi Yamaura, Satoko Yoshikura, Douglas W. Yu, Andrey S. Zaitsev, Juliane Zeidler, Fasheng Zou, Ben Collen, Rob M. Ewers, Georgina M. Mace, Drew W. Purves, Jörn P. W. Scharlemann, Andy Purvis

**Affiliations:** ^1^ Department of Life SciencesNatural History MuseumLondonUK; ^2^ United Nations Environment Programme World Conservation Monitoring CentreCambridgeUK; ^3^ Department of Genetics, Evolution and EnvironmentCentre for Biodiversity and EnvironmentResearchUniversity College LondonLondonUK; ^4^ Department of Life SciencesImperial College LondonAscotUK; ^5^ Imperial College LondonSouth KensingtonLondonUK; ^6^ Department of ZoologyCambridge UniversityCambridgeUK; ^7^ Frankfurt Zoological SocietyAfrica Regional OfficeArushaTanzania; ^8^ Science and Solutions for a Changing Planet DTP and the Department of Life SciencesImperial College LondonSouth KensingtonLondonUK; ^9^ Centre d’étude de la forêt.Université LavalLavalQCCanada; ^10^ School of Life SciencesUniversity of SussexBrightonUK; ^11^ School of Biological and Chemical SciencesQueen Mary University of LondonLondonUK; ^12^ School of Biological and Ecological SciencesUniversity of StirlingStirlingUK; ^13^ School of Biological SciencesRoyal Holloway University of LondonEgham, SurreyUK; ^14^ Department of Animal and Plant SciencesUniversity of SheffieldWestern BankSheffieldUK; ^15^ School of EnvironmentNatural Resources and GeographyBangor UniversityBangorGwyneddUK; ^16^ University College LondonLondonUK; ^17^ School of Biological SciencesQueen's University BelfastBelfastUK; ^18^ Institute of Biological and Environmental SciencesUniversity of AberdeenAberdeenUK; ^19^ Evolutionary Ecology GroupUniversity of AntwerpAntwerpBelgium; ^20^ Nees Institute for Plant BiodiversityUniversity of BonnBonnGermany; ^21^ Wildlife and Range Management DepartmentFaculty of Renewable Natural Resources (FRNR)College of Agriculture and Natural Resources (CANR)Kwame Nkrumah University of Science and Technology (KNUST)KumasiGhana; ^22^ SAVE THE FROGS! GhanaAdum‐KumasiGhana; ^23^ Escuela de BiologíaUniversidad de Costa RicaSan JoséCosta Rica; ^24^ Laboratorio Ecotono‐CRUBUniversidad Nacional del Comahue and INIBIOMARío NegroArgentina; ^25^ Departamento de BotánicaFacultad de FarmaciaUniversidad de ValenciaBurjassot, ValenciaSpain; ^26^ Marine LaboratorySilliman University‐Angelo King Center for Research and Environmental ManagementSilliman UniversityDumaguete CityPhilippines; ^27^ Department of Soil and Water ConservationCSIC‐Centro de Edafología y Biología Aplicada del SeguraMurciaSpain; ^28^ INRAUR 0980 SAD‐PaysageRennes CedexFrance; ^29^ INRAUMR 1201 DYNAFORCastanet Tolosan CedexFrance; ^30^ HUTAN – Kinabatangan Orang‐utan Conservation ProgrammeKota KinabaluMalaysia; ^31^ Borneo FuturesKota KinabaluMalaysia; ^32^ CSIRO Land & Water FlagshipWinnellieNTAustralia; ^33^ Museo de ZoologíaFacultad de CienciasUniversidad Nacional Autónoma de MéxicoMéxico D.F.Mexico; ^34^ Colección de TejidosInstituto de Investigación de Recursos Biológicos Alexander von HumboldtValle del CaucaColombia; ^35^ Biology DepartmentUniversidad del ValleCaliColombia; ^36^ Instituto de Investigaciones en Ecosistemas y SustentabilidadUniversidad Nacional Autónoma de MéxicoMoreliaMexico; ^37^ College of Science, Engineering & HealthRMIT UniversityMelbourneVic.Australia; ^38^ UCL Department of GeographyUniversity College LondonLondonUK; ^39^ Biodiversity UnitInstitute of BioscienceUniversiti Putra MalaysiaSerdangMalaysia; ^40^ Faculty of ForestryUniversiti Putra MalaysiaSerdangMalaysia; ^41^ Departamento de Biodiversidad y GenéticaInstituto de Investigaciones Biológicas Clemente EstableMontevideoUruguay; ^42^ Forest & Nature LabDepartment of Forest and Water ManagementGhent UniversityGontrodeBelgium; ^43^ Terrestrial Ecology UnitDepartment of BiologyGhent UniversityGhentBelgium; ^44^ UFR Science de la NatureUniversité Naangui AbrogouaAbidjanIvory Coast; ^45^ Centre Suisse de Recherches Scientifiques en Côte d'IvoireAbidjanIvory Coast; ^46^ MTA Centre for Ecological ResearchVácrátótHungary; ^47^ University of Washington TacomaTacomaWAUSA; ^48^ Northern Hardwoods Research InstituteEdmundstonNBCanada; ^49^ Lancaster Environment CentreLancaster UniversityLancasterUK; ^50^ MCT/Museu Paraense Emílio GoeldiBelémBrazil; ^51^ AgResearch LimitedInvermay Agricultural CentrePuddle Alley, MosgielNew Zealand; ^52^ Centre for Functional EcologyDepartment of Life SciencesUniversity of CoimbraCoimbraPortugal; ^53^ COT (Tuscan Ornithological Society)LivornoItaly; ^54^ Smithsonian Tropical Research InstituteBalboaAnconPanama CityRepublic of Panama; ^55^ AgroecologyDepartment of Crop SciencesGeorg‐August UniversityGöttingenGermany; ^56^ BiosciencesSchool of Science & TechnologyNottingham Trent UniversityClifton, NottinghamUK; ^57^ University of BirminghamEdgbaston, BirminghamUK; ^58^ Section of Conservation BiologyDepartment of Environmental SciencesUniversity of BaselBaselSwitzerland; ^59^ Department of Biological SciencesUniversity of AlbertaEdmontonABCanada; ^60^ CIBIO/InBioCentro de Investigação em Biodiversidade e Recursos GenéticosUniversidade do PortoVairãoPortugal; ^61^ Faculty of Sustainable AgricultureUniversiti Malaysia SabahSandakanMalaysia; ^62^ The Swedish University of Agricultural SciencesThe Swedish Biodiversity CentreUppsalaSweden; ^63^ Institute for Tropical Biology and ConservationUniversiti Malaysia Sabah, Jalan UMSKota KinabaluMalaysia; ^64^ School of GeosciencesUniversity of EdinburghEdinburghUK; ^65^ Department of Zoology & Environmental ScienceGurukula Kangri UniversityHaridwarIndia; ^66^ Durrell Institute of Conservation and Ecology (DICE)School of Anthropology and ConservationUniversity of KentCanterburyUK; ^67^ Iwokrama International Centre for Rainforest Conservation and DevelopmentGeorgetownGuyana; ^68^ Department of Ecology‐Animal EcologyFaculty of BiologyPhilipps‐Universität MarburgMarburgGermany; ^69^ Compliance Services InternationalPentlands Science ParkPenicuik, EdinburghUK; ^70^ Centre for Agri‐Environmental ResearchSchool of Agriculture, Policy and DevelopmentUniversity of ReadingReadingUK; ^71^ School for the Training of Wildlife Specialists GarouaGarouaCameroon; ^72^ Department of ForestryFaculty of Agronomy and Agricultural SciencesUniversity of DschangDschangCameroon; ^73^ Mater Natura – Instituto de Estudos AmbientaisCuritibaBrazil; ^74^ CBS Fungal Biodiversity Centre (CBS‐KNAW)UtrechtThe Netherlands; ^75^ Senckenberg Biodiversity and Climate Research Centre (BiK‐F)Frankfurt am MainGermany; ^76^ Institute for Ecology, Evolution & DiversityGoethe University FrankfurtBiologicum, Frankfurt am MainGermany; ^77^ School of Land and FoodUniversity of TasmaniaSandy BayTas.Australia; ^78^ Departamento de Ciências AgráriascE3c – Centre for Ecology, Evolution and Environmental Changes/Azorean Biodiversity Group and Universidade dos AçoresAngra do Heroísmo, AçoresPortugal; ^79^ Instituto Nacional de Pesquisas da AmazôniaManausBrazil; ^80^ Environment and Climate Change Canada, Science & Technology BranchCarleton UniversityOttawaONCanada; ^81^ Unité Mixte de Recherche Contrôle des Maladies Animales Exotiques et EmergentesCentre de Coopération Internationale en Recherche Agronomique pour le Développement (CIRAD)MontpellierFrance; ^82^ Unité Mixte de Recherche 1309 Contrôle des Maladies Animales Exotiques et EmergentesInstitut national de la recherche agronomique (INRA)MontpellierFrance; ^83^ Departamento de ZoologiaInstituto de BiociênciasUniversidade de São PauloSão PauloBrazil; ^84^ Human Environment Systems CenterBoise State UniversityBoiseIDUSA; ^85^ School of Science and the EnvironmentManchester Metropolitan UniversityManchesterUK; ^86^ Universidade de Évora – ICAAMÉvoraPortugal; ^87^ Natural Parks Technical OfficeDiputació de BarcelonaBarcelonaSpain; ^88^ Natural History Museum of BarcelonaBarcelona, CataloniaSpain; ^89^ Swedish University of Agricultural SciencesSouthern Swedish Forest Research CentreAlnarpSweden; ^90^ Department of EntomologyPurdue UniversityWest LafayetteINUSA; ^91^ Department of Natural Resource SciencesMcGill UniversitySte‐Ann‐de‐BellevueQCCanada; ^92^ Alterra, part of Wageningen University and ResearchRB WageningenThe Netherlands; ^93^ Departamento de Ciências da VidaCentro de Ecologia FuncionalUniversidade de CoimbraCoimbraPortugal; ^94^ Departamento de Biologia VegetalInstituto de BiologiaUniversidade Estadual de CampinasCampinasBrazil; ^95^ Department of BotanySchool of Natural SciencesTrinity College DublinDublin 2Ireland; ^96^ Institute for Environmental SciencesUniversity Koblenz‐LandauLandauGermany; ^97^ Departamento de ZoologiaInstituto de BiociênciasUniversidade de São PauloSão PauloBrazil; ^98^ Departamento de BiologíaGrupo de investigación en BiologíaEcología y Manejo de HormigasSección de EntomologíaUniversidad del ValleCaliColombia; ^99^ Department of BiologyFederal University of Santa Maria, CCNESanta MariaBrazil; ^100^ Nicholas School of the EnvironmentDuke UniversityDurhamNCUSA; ^101^ Department of Ecology and Animal BiologyFaculty of SciencesUniversity of VigoVigoSpain; ^102^ Department of EntomologyUniversity of IllinoisUrbanaILUSA; ^103^ Program in Ecology, Evolution and Conservation BiologyUniversity of IllinoisUrbanaILUSA; ^104^ Museu de Zoologia da Universidade de São PauloSão PauloBrazil; ^105^ Departamento de Biología (Botánica)Facultad de CienciasUniversidad Autonoma de MadridMadridSpain; ^106^ Finnish Museum of Natural HistoryUniversity of HelsinkiHelsinkiFinland; ^107^ Parks and CountrysideBracknell Forest CouncilBracknellUK; ^108^ Soil Biodiversity GroupLife Sciences DepartmentNatural History MuseumLondonUK; ^109^ Museu de Zoologia da Universidade de São PauloSão PauloBrazil; ^110^ Laboratório de Ecologia Aplicada à ConservaçãoUniversidade Estadual de Santa CruzIlhéusBrazil; ^111^ Instituto de Biotecnologia y Ecologia Aplicada (INBIOTECA)Universidad VeracruzanaXalapaMexico; ^112^ Centro Agronómico Tropical de Investigación y Enseñanza (CATIE)Tropical Agricultural Research and Higher Education CenterTurrialbaCosta Rica; ^113^ Department of Quantitative Methods and Information SystemsFaculty of AgronomyUniversity of Buenos AiresBuenos AiresArgentina; ^114^ Applied Ecological Services, Inc.Prior LakeMNUSA; ^115^ Normandie UnivEA 1293 ECODIV‐RouenSFR SCALEUFR Sciences et TechniquesMont Saint Aignan CedexFrance; ^116^ MC‐ConsultSorøDenmark; ^117^ Institute of Biological and Environmental SciencesUniversity of AberdeenAberdeenUK; ^118^ Department of BiologyCESAMUniversidade de AveiroAveiroPortugal; ^119^ Dipartimento di BiologiaUniversità degli Studi di MilanoMilanoItaly; ^120^ Sustainability Research InstituteUniversity of East LondonLondonUK; ^121^ Centre of Excellence for Environmental DecisionsSchool of Plant BiologyUniversity of Western AustraliaNedlandsWAAustralia; ^122^ School of Veterinary and Life SciencesMurdoch UniversityMurdochWAAustralia; ^123^ Grupo Ecología de Artrópodos y Manejo de PlagasEl Colegio de la Frontera SurTapachulaMexico; ^124^ CSIRO Land and Water FlagshipCanberraACTAustralia; ^125^ Dipartimento di BiologiaUniversità di Napoli Federico IINapoliItaly; ^126^ Wildlife Conservation Research UnitDepartment of ZoologyUniversity of OxfordRecanati‐Kaplan CentreTubneyUK; ^127^ Programa de Pós‐Graduação em EcologiaUniversidade Federal de Santa CatarinaFlorianópolisBrazil; ^128^ Sustainability Research InstituteSchool of Earth and EnvironmentUniversity of LeedsLeedsUK; ^129^ British Trust for OrnithologyStirling, UK; ^130^ Thünen Institute of BiodiversityBraunschweigGermany; ^131^ Scarab Research GroupDepartment of Zoology & EntomologyUniversity of PretoriaHatfieldSouth Africa; ^132^ Durrell Wildlife Conservation TrustTrinityJersey; ^133^ Center for International Forestry ResearchBogorIndonesia; ^134^ Kwata NGOCayenneFrench Guiana; ^135^ CIRADUMR SystemMontpellierFrance; ^136^ ICRAFRegional Office for Latin AmericaLimaPeru; ^137^ UPSINPLaboratoire Écologie Fonctionnelle et EnvironnementUniversité de ToulouseToulouseFrance; ^138^ CNRS – UMR 5245EcolabToulouseFrance; ^139^ CNRS – UMR 8172Écologie des Forêts de GuyaneKourou cedexFrance; ^140^ CNRS – UMR 7206 (retired) CNRS/MNHNParisFrance; ^141^ Department of Landscape EcologyInstitute of Natural Resource ConservationKiel UniversityKielGermany; ^142^ Department of Biology, Nature ConservationUniversity MarburgMarburgGermany; ^143^ Institute of Integrative BiologyETH ZürichZürichSwitzerland; ^144^ Post Graduate Program in Wildlife Biology and ConservationNational Centre for Biological SciencesBangaloreIndia; ^145^ Wildlife Conservation Society (India Program)Centre for Wildlife StudiesBangaloreIndia; ^146^ Instituto de Investigaciones Agropecuarias – INIA – CRI – KampenaikePunta ArenasChile; ^147^ Programa de BiologíaUniversidad del AtlánticoBarranquillaColombia; ^148^ Applied EntomologyETH ZürichZürichSwitzerland; ^149^ Unit for Environmental Sciences and ManagementNorth‐West UniversityPotchefstroomSouth Africa; ^150^ Department of Ecosystem ModellingBüsgen‐InstituteGeorg‐August‐University of GöttingenGöttingenGermany; ^151^ INRAUMR 1213 HerbivoresSaint‐Genès ChampanelleFrance; ^152^ Institute of ZoologyZoological Society of London, Regents ParkLondonUK; ^153^ Department of Ecology and Environmental ScienceUmeå UniversityUmeåSweden; ^154^ Department of Wildlife, Fish and Environmental StudiesSwedish University of Agricultural SciencesUmeaSweden; ^155^ Centre for Biological SciencesUniversity of SouthamptonSouthamptonUK; ^156^ MTA‐ELTE‐MTM Ecology Research GroupHungarian Academy of Sciencesc/o Biological InstituteEötvös Lóránd UniversityBudapestHungary; ^157^ Hungarian Natural History MuseumBudapestHungary; ^158^ Institute for Environmental SciencesUniversity of Koblenz‐LandauLandauGermany; ^159^ Department of Conservation Ecology and EntomologyStellenbosch UniversityMatielandSouth Africa; ^160^ Centre for Invasion BiologyStellenbosch UniversityMatielandSouth Africa; ^161^ CE3C – Centre for Ecology, Evolution and Environmental ChangesFaculdade de CiênciasUniversidade de LisboaLisboaPortugal; ^162^ Associação Monte PicoMonte CaféMé ZóchiSão Tomé and Príncipe; ^163^ Kew GardensWakehurstArdingly, Haywards Heath, SussexUK; ^164^ Wild AsiaUpper PenthouseWisma RKTKuala LumpurMalaysia; ^165^ Conservation EcologyFaculty of BiologyPhilipps‐Universität MarburgMarburgGermany; ^166^ Institute of EntomologyBiology Centre of Academy of Sciences Czech RepublicČeské BudějoviceCzech Republic; ^167^ Institute for Tropical Biology and ConservationUniversiti Malaysia SabahKota KinabaluMalaysia; ^168^ Dipartimento di Scienze VeterinarieUniversità di PisaPisaItaly; ^169^ Swedish University of Agricultural SciencesAlnarpSweden; ^170^ Department of Biological SciencesUniversity of QueenslandSt LuciaQldAustralia; ^171^ Queensland Herbarium (DSITIA)ToowongQldAustralia; ^172^ School of SustainabilityArizona State UniversityTempeAZUSA; ^173^ Department of BiologyTrent UniversityPeterboroughONCanada; ^174^ Laboratoire d'Ecologie Alpine (LECA)Université Grenoble AlpesGrenobleFrance; ^175^ Institute of Biology Bucharest of Romanian AcademyBucharestRomania; ^176^ Universidade Federal de Pernambuco – UFPECidade UniversitariaRecifeBrazil; ^177^ Tarla Bitkileri Merkez Araştırma EnstitüsüYenimahalle‐AnkaraTurkey; ^178^ School of Forest Resources and Environmental ScienceMichigan Technological UniversityHoughtonMIUSA; ^179^ Department of Animal Ecology and Tropical BiologyBiocenterUniversity of WürzburgWürzburgGermany; ^180^ Department of Plant SciencesUniversity of CaliforniaDavisCAUSA; ^181^ Department of Soil and Crop SciencesColorado State UniversityFort CollinsCOUSA; ^182^ IRD‐UMR 208 PALOC IRD/MNHNParisFrance; ^183^ Department of Community EcologyUFZHelmholtz Centre for Environmental ResearchHalleGermany; ^184^ Department of Natural Resource SciencesThompson Rivers UniversityKamloopsBCCanada; ^185^ Institute for Biodiversity and Ecosystem Dynamics (IBED)University of AmsterdamGE AmsterdamThe Netherlands; ^186^ PanEco/Yayasan Ekosistem LestariSumatran Orangutan Conservation ProgrammeMedanIndonesia; ^187^ Programa de Pós Graduação em EcologiaUniversidade de BrasíliaBrasília, Distrito FederalBrazil; ^188^ IDEA Consultants Inc.Okinawa Branch OfficeNahaJapan; ^189^ Biocentre GrindelUniversity of HamburgHamburgGermany; ^190^ Departamento de ZoologíaFacultad de Ciencias Naturales y OceanográficasUniversidad de ConcepciónConcepciónChile; ^191^ Departamento de Planificación TerritorialFacultad de Ciencias AmbientalesCentro EULA‐ChileUniversidad de ConcepciónConcepciónChile; ^192^ Hopkirk InstituteMassey UniversityPalmerston NorthNew Zealand; ^193^ Seed Consulting ServicesAdelaideSAAustralia; ^194^ Environmental Futures Research InstituteGriffith UniversityBrisbaneQldAustralia; ^195^ Barbara Hardy InstituteUniversity of South AustraliaMawson LakesSAAustralia; ^196^ Jiangsu Key Laboratory for Bioresources of Saline SoilsYancheng Teachers UniversityYanchengChina; ^197^ Département des sciences biologiquesCentre d’études de la forêt Université du Québec à Montréal Succursale Centre‐villeMontréalQCCanada; ^198^ AgResearchRuakura Research CentreHamiltonNew Zealand; ^199^ Ecologia Aplicada/Applied EcologyUniversidade Sagrado Coração (USC)BauruBrazil; ^200^ Department of Ecology and Evolutionary BiologyUniversity of TorontoTorontoONCanada; ^201^ DIFARUniversity of GenovaGenovaItaly; ^202^ Tel Aviv UniversityTel AvivIsrael; ^203^ World Wildlife Fund, Inc. (WWF) GuianasParamariboSuriname; ^204^ Rubenstein School of Natural ResourcesUniversity of VermontBurlingtonVTUSA; ^205^ Astron Environmental ServicesEast PerthWAAustralia; ^206^ Department of Environment and AgricultureCurtin UniversityPerthWAAustralia; ^207^ Centre de Biologie pour la Gestion des Populations (CBGP)INRAIRDCIRADSUPAGROMontferrier‐sur‐Lez cedexFrance; ^208^ Department of ZoologyUniversity of OxfordOxfordUK; ^209^ Department of Biological SciencesMount Holyoke CollegeSouth HadleyMAUSA; ^210^ China International Engineering Consulting CorporationHaidian DistrictBeijingChina; ^211^ CREAFCerdanyola del Vallès, CataloniaSpain; ^212^ Departamento de Ciencias Ambientales y Recursos Naturales RenovablesFacultad de Ciencias AgronómicasUniversidad de ChileLa PintanaChile; ^213^ Grupos de FaunaInstituto amazónico de investigaciones científicas Sinchi.BogotáColombia; ^214^ Biodiversity, Evolution and Ecology of Plants (BEE)Biocentre Klein Flottbek and Botanical GardenUniversity of HamburgHamburgGermany; ^215^ School of Biological ScienceUniversity of PlymouthPlymouthUK; ^216^ Friday HarborWAUSA; ^217^ International University of Malaya‐Wales, Jalan Tun IsmailKuala LumpurMalaysia; ^218^ Department of Wildlife ManagementSokoine University of AgricultureMorogoroTanzania; ^219^ The Xerces Society for Invertebrate ConservationPortlandORUSA; ^220^ Animal & Environment Research GroupDepartment of Life SciencesAnglia Ruskin UniversityCambridgeUK; ^221^ Walter Sisulu UniversityMthatha, TranskeiSouth Africa; ^222^ Centre for African Conservation EcologyNelson Mandela Metropolitan UniversityPort ElizabethSouth Africa; ^223^ College of Natural SciencesBangor UniversityBangor, GwyneddUK; ^224^ Natural Resources CanadaCanadian Forest ServiceLaurentian Forestry CentreQuébecQCCanada; ^225^ Department of Arctic and Marine BiologyUniversity of TromsøTromsøNorway; ^226^ PantheraNew YorkNYUSA; ^227^ Universidad Nacional Experimental de GuayanaPuerto OrdazVenezuela; ^228^ Richard Gilder Graduate SchoolAmerican Museum of Natural HistoryNew YorkNYUSA; ^229^ AgroscopeZürichSwitzerland; ^230^ Corporación Sentido NaturalBogotáColombia; ^231^ Earth and Atmospheric Sciences DepartmentUniversity of AlbertaEdmontonABCanada; ^232^ State Museum of Natural History Karlsruhe (SMNK)BiosciencesKarlsruheGermany; ^233^ Museum für Naturkunde – Leibniz Institute for Evolution and Biodiversity ScienceBerlinGermany; ^234^ University of Technology SydneySydneyNSWAustralia; ^235^ University of New BrunswickFrederictonNBCanada; ^236^ Department of EcologyFaculty of Veterinary ScienceSZIE UniversityBudapestHungary; ^237^ Department of EcologyUniversity of DebrecenDebrecenHungary; ^238^ Department of Ecology, Environment and Plant SciencesStockholm UniversityStockholmSweden; ^239^ Instituto de Investigaciones y Recursos Biológicos Alexander von HumboldtBogotá, Colombia; ^240^ Institute of Natural and Environmental SciencesUniversity of HyogoHyogoJapan; ^241^ Hiroshima UniversityLeading‐programHigashihiroshimaKagamiyamaJapan; ^242^ Instituto de Investigaciones en Ecosistemas y SustentabilidadUniversidad Nacional Autónoma de MéxicoMoreliaMéxico C.P.Mexico; ^243^ Department of Animal EcologyJustus‐Liebig‐UniversityGiessenGermany; ^244^ Escuela de BiologiaUniversidad de Costa RicaSan PedroCosta Rica; ^245^ Biodiversity and Environmental SustainabilityRohiniIndia; ^246^ Department of Environmental StudiesShivaji College (University of Delhi)New DelhiIndia; ^247^ Department of EcologySwedish University of Agricultural SciencesUppsalaSweden; ^248^ School of Biological SciencesUniversiti Sains MalaysiaMindenMalaysia; ^249^ Yukon Department of EnvironmentWhitehorseYTCanada; ^250^ Nature Conservation FoundationMysore, India; ^251^ Cologne BiocenterZoological InstituteUniversity of CologneKölnGermany; ^252^ Department of Environmental & Natural Resources ManagementUniversity of PatrasAgrinioGreece; ^253^ Centre for Tropical Environmental and Sustainability Science (TESS) & College of Marine and Environmental SciencesJames Cook UniversityCairnsQldAustralia; ^254^ School of Science and TechnologyPacific Adventist UniversityPort MoresbyPapua New Guinea; ^255^ Unit for Environmental Sciences and ManagementNorth‐West UniversityPotchefstroomSouth Africa; ^256^ Department of Systematic and Evolutionary BotanyUniversity of ZürichZürichSwitzerland; ^257^ Department of Ecology and Evolutionary Biology and Department of Geography and PlanningUniversity of TorontoTorontoONCanada; ^258^ The Wilderness & Wildlife Conservation TrustColomboSri Lanka; ^259^ School of Biological SciencesPlymouth UniversityPlymouthUK; ^260^ Institute of Ecology and EvolutionUniversity of BernBernSwitzerland; ^261^ Section EnvironnementDéveloppement durable et TerritoireDivision Environnement et TerritoireBundesamt für StatistikNeuchâtelSwitzerland; ^262^ School of Forest SciencesUniversity of Eastern FinlandJoensuuFinland; ^263^ Institute of Ecology, FB2University of BremenBremenGermany; ^264^ Université Peleforo Gon CoulibalyKorhogoIvory Coast; ^265^ Station d'Ecologie de LamtoN'DouciIvory Coast; ^266^ Theoretical Evolutionary Ecology GroupDepartment of Animal Ecology and Tropical BiologyBiocenterUniversity of WürzburgWürzburgGermany; ^267^ Wildlife Conservation Society‐IndiaNational Centre for Biological SciencesBangaloreIndia; ^268^ Nature Science InitiativeDehradunIndia; ^269^ Department of Environmental Science, Policy, and ManagementUniversity of CaliforniaBerkeleyCAUSA; ^270^ School of BioSciencesUniversity of MelbourneMelbourneVic.Australia; ^271^ School of Agricultural, Forest and Food Sciences HAFLBern University of Applied SciencesZollikofenSwitzerland; ^272^ Swiss Federal Institute for ForestSnow and Landscape Research WSLBirmensdorfSwitzerland; ^273^ Instituto Nacional de Tecnología AgropecuariaEEA BarilocheBarilocheArgentina; ^274^ Department of BiologyPennsylvania State UniversityUniversity ParkPAUSA; ^275^ National AviaryAllegheny Commons WestPittsburghPAUSA; ^276^ Centre for Tropical Environmental and Sustainability SciencesCollege of Marine and Environmental ScienceJames Cook UniversityCairnsQldAustralia; ^277^ Université Pierre‐et‐Marie‐CurieParisFrance; ^278^ Institute of Ecology and Environmental SciencesParisFrance; ^279^ INRAUR 406 Abeilles et EnvironnementAvignonFrance; ^280^ Department of BiologySan Francisco State UniversitySan FranciscoCAUSA; ^281^ Laboratoire de diagnostic en phytoprotectionMinistère de l'agriculture, des pêcheries et de l'alimentation du QuébecVille de QuébecQCCanada; ^282^ Research Unit Terrestrial EcologyGhent UniversityGhentBelgium; ^283^ Laboratorio de Recursos AgroforestalesCentro Austral de Investigaciones Científicas (CADIC)Consejo Nacional de Investigaciones Científicas y Técnicas (CONICET)UshuaiaArgentina; ^284^ School of BiosciencesUniversity of MelbourneParkvilleVic.Australia; ^285^ Purchase College (State University of New York)PurchaseNYUSA; ^286^ Institute of Applied EcologyChinese Academy of SciencesShenyangChina; ^287^ School of Biological SciencesUniversity of CanterburyChristchurchNew Zealand; ^288^ The James Hutton InstituteAberdeenUK; ^289^ College of Resources and Environmental SciencesChina Agricultural UniversityBeijingChina; ^290^ Carste Ciência e Meio AmbienteFloresta, Belo HorizonteBrazil; ^291^ TEHO LaboratoryInstitiute of BiologyUniversity of AntioquiaMedellínColombia; ^292^ International Center for Agricultural Research in the Dry Areas (ICARDA)Amman OfficeAmmanJordan; ^293^ Animal and Rangeland Sciences DepartmentOregon State UniversityCorvallisORUSA; ^294^ Department of AgroecologyFlakkebjerg Research CentreAarhus UniversitySlagelseDenmark; ^295^ Department of Agroforestry Technology and Science and GeneticsSchool of Advanced Agricultural EngineeringCastilla La Mancha UniversityAlbaceteSpain; ^296^ Unidad Académica de TurismoCoordinación de Investigación y PosgradoUniversidad Autónoma de NayaritTepicMexico; ^297^ Centro de Investigaciones TropicalesUniversidad VeracruzanaXalapaMexico; ^298^ Graduate School of Agricultural ScienceKobe UniversityKobeJapan; ^299^ Department of EcologyUniversity of DebrecenDebrecenHungary; ^300^ Center for Conservation InnovationSan Jose Tagaytay CityPhilippines; ^301^ Biology DepartmentDe La Salle UniversityManilaPhilippines; ^302^ The New Zealand Institute for Plant & Food Research LimitedAucklandNew Zealand; ^303^ National Museums of KenyaNairobiKenya; ^304^ Center for Macroecology, Evolution and ClimateNatural History Museum of DenmarkUniversity of CopenhagenCopenhagen ØDenmark; ^305^ Red de Biología y Conservación de VertebradosInstituto de Ecología A.C.XalapaMexico; ^306^ Department of GeographyUniversity of BergenBergenNorway; ^307^ Department of GeographyUniversity of Wisconsin‐MadisonMadisonWIUSA; ^308^ School of BiologyUniversity of LeedsLeedsWest YorkshireUK; ^309^ Marshall Agroecology LtdBartonWinscombeUK; ^310^ Universidad Nacional de Colombia, Ciudad UniversitariaBogotáColombia; ^311^ Basque Centre for Climate Change – BC3BilbaoSpain; ^312^ School of Biological SciencesThe University of QueenslandBrisbaneQldAustralia; ^313^ Associate of Arts ProgramUniversity of Delaware – WilmingtonWilmingtonDEUSA; ^314^ Department of Entomology and Wildlife EcologyUniversity of DelawareNewarkDEUSA; ^315^ Department of EntomologyUniversity of CaliforniaRiversideCAUSA; ^316^ Centre for Mined Land RehabilitationThe University of QueenslandBrisbaneQldAustralia; ^317^ Departamento de Biogeografía y Cambio GlobalMuseo Nacional de Ciencias Naturales (CSIC)MadridSpain; ^318^ Ecology and Evolutionary BiologyUniversity of ConnecticutStorrsCTUSA; ^319^ Museo de Historia Natural “Vera Alleman Haeghebaert”Universidad Ricardo PalmaLima 33Peru; ^320^ Centro Iberoamericano de la Biodiversidad (CIBIO)Universidad de AlicanteAlicanteSpain; ^321^ Department of EcologySwedish University of Agricultural Sciences, Grimsö Wildlife Research StationRiddarhyttanSweden; ^322^ Rainforest AllianceNew YorkNYUSA; ^323^ Department of Natural ResourcesCornell UniversityIthacaNYUSA; ^324^ Department of Natural Resources & Environmental SciencesUniversity of IllinoisUrbanaILUSA; ^325^ Universidad Industrial de SantanderBucaramangaColombia; ^326^ School of Plant BiologyUniversity of Western AustraliaCrawleyWAAustralia; ^327^ Lab. EcotonoINIBIOMA (Universidad Nacional del Comahue‐CONICET)BarilocheArgentina; ^328^ Botany DepartmentNational Museums of KenyaNairobiKenya; ^329^ Department of Wildlife ManagementUniversity of EldoretEldoretKenya; ^330^ Department of Biology and EcologyFaculty of SciencesUniversity of Novi SadNovi SadSerbia; ^331^ School of Biological SciencesUniversiti Sains MalaysiaPenangMalaysia; ^332^ El Colegio de la Frontera SurEcología Evolutiva y ConservaciónSan Cristóbal de las CasasMexico; ^333^ Nature KenyaNairobiKenya; ^334^ WWFWashingtonDCUSA; ^335^ Independent Research ScholarNew DelhiIndia; ^336^ Avian Diversity and Bioacoustic LabDepartment of ZoologyGurukula Kangri UniversityHaridwarIndia; ^337^ Graduate School of Bioagricultural SciencesNagoya UniversityNagoyaJapan; ^338^ Key Laboratory of Tropical Forest EcologyXishuangbanna Tropical Botanical GardenChinese Academy of SciencesMenglunChina; ^339^ Environmental Futures Research Institute, and Griffith School of EnvironmentGriffith UniversityNathanBrisbaneQldAustralia; ^340^ College of Bioresource ScienceNihon UniversityFujisawaJapan; ^341^ Forestry and Forest Products Research InstituteTsukubaJapan; ^342^ Laboratorio de Investigaciones en Abejas (Departamento de Biología)Universidad Nacional de ColombiaBogotáColombia; ^343^ Laboratorio de Información GeográficaEl Colegio de la Frontera Sur (ECOSUR)San Cristóbal de las CasasMexico; ^344^ CMRPZ – I.E. Plaza BonitaSan Andrés de Sotavento (Córdoba)Colombia; ^345^ BirdLife International – Africa Partnership SecretariatNairobiKenya; ^346^ Ornithology SectionNational Museums of KenyaNairobiKenya; ^347^ Department of ZoologyUniversity of British ColumbiaVancouverBCCanada; ^348^ Institut de Systématique, Évolution, BiodiversitéISYEB – UMR 7205 – CNRS, MNHN, UPMC, EPHEMuséum national d'Histoire naturelleSorbonne UniversitésParisFrance; ^349^ Department of Biology/BiodiversityLund UniversityLundSweden; ^350^ Department of BiosciencesUniversity of HelsinkiHelsinkiFinland; ^351^ Department of Environmental SciencesUniversity of HelsinkiHelsinkiFinland; ^352^ School of BiologyThe University of NottinghamUniversity ParkNottinghamUK; ^353^ Laboratorio de Zoología y Ecología Acuática – LAZOEAUniversidad de Los AndesBogotáColombia; ^354^ School of ForestryUniversity of CanterburyChristchurchNew Zealand; ^355^ BIO‐DiverseBonnGermany; ^356^ Department of Wildlife, Fish and Conservation BiologyUniversity of California, DavisDavisCAUSA; ^357^ IUCN‐Centre for Mediterranean CooperationCampanillas, MálagaSpain; ^358^ Oxford University Centre for the EnvironmentUniversity of OxfordOxfordUK; ^359^ Natural Resources and the EnvironmentCSIRStellenboschSouth Africa; ^360^ Plant Conservation UnitBiological SciencesUniversity of Cape TownRondeboschSouth Africa; ^361^ International Programme Office (IPO)Vice Chancellor's OfficeKwame Nkrumah University of Science and Technology (KNUST)KumasiGhana; ^362^ Naturschutz – Planung und BeratungWiesendangenSwitzerland; ^363^ Department of Wildlife and Range ManagementKwame Nkrumah University of Science and TechnologyKumasiGhana; ^364^ Forestry Research Institute of GhanaKumasiGhana; ^365^ Department of Animal & Environmental BiologyUniversity of BeninBenin CityNigeria; ^366^ Department of Genetics, Evolution and EnvironmentUniversity College LondonLondonUK; ^367^ The Royal Society for the Protection of Birds (RSPB)Sandy, BedfordshireUK; ^368^ Laboratorio de Ecología del PaisajeFacultad de Ciencias ForestalesUniversidad de ConcepciónConcepciónChile; ^369^ Indian Institute of ScienceBangaloreIndia; ^370^ Laboratorio EcotonoCONICET–INIBIOMAUniversidad Nacional del ComahueBarilocheArgentina; ^371^ Laboratorio de Investigaciones en AbejasLABUNUniversidad Nacional de ColombiaBogotá D.C.Colombia; ^372^ Lancaster Environment CentreLancaster UniversityLancasterUK; ^373^ Universidade Federal do Pará (UFPA)Núcleo de Altos Estudos Amazonicos (NAEA)BelémBrazil; ^374^ German Centre for Integrative Biodiversity Research (iDiv)Halle‐Jena‐LeipzigLeipzigGermany; ^375^ Department of Plant Biology and EcologyFaculty of Science and TechnologyUniversity of the Basque CountryLeioaSpain; ^376^ IKERBASQUE. Basque Foundation for ScienceBilbaoSpain; ^377^ Instituto de Diversidad y Ecología Animal (IDEA, CONICET‐UNC) and Centro de Zoología AplicadaFCEFyNUniversidad Nacional de CórdobaCórdobaArgentina; ^378^ IRDUMR AMAPTA A51/PS2Montpellier cedex 05France; ^379^ French Institute of PondicherryUMIFRE 21 CNRS‐MAEEPuducherryIndia; ^380^ School of Environmental SciencesUniversity of East AngliaNorwichUK; ^381^ National Institute of Agricultural Technology (INTA)Río GallegosArgentina; ^382^ National University of Southern Patagonia (UNPA)Río GallegosArgentina; ^383^ National Commission of Scientist Research and Technology (CONICET)Buenos Aires, Argentina; ^384^ Laboratory of Biogeography & EcologyDepartment of GeographyUniversity of the AegeanMytileneGreece; ^385^ Department of Animal Ecology and Tropical BiologyBiocenterUniversity of WürzburgWürzburgGermany; ^386^ University of CambridgeCambridgeUK; ^387^ Conservation Science GroupDepartment of ZoologyUniversity of CambridgeCambridgeUK; ^388^ Systematics and Evolution LaboratoryDepartment of BiologyWestern Kentucky UniversityBowling GreenKYUSA; ^389^ Department of Natural Resource Ecology and ManagementIowa State UniversityAmesIAUSA; ^390^ Facultad de Recursos NaturalesEscuela de Ciencias AmbientalesLaboratorio de Planificación TerritorialUniversidad Católica de TemucoTemucoChile; ^391^ Biología y Conservación de VertebradosInstituto de Ecología A.C.El Haya, XalapaMexico; ^392^ Universitat Autònoma de BarcelonaCerdanyola del VallèsSpain; ^393^ Laboratorio de Entomología EcológicaDepartamento de BiologíaFacultad de CienciasUniversidad de La SerenaLa SerenaChile; ^394^ Albertine Rift ProgramWildlife Conservation SocietyKampalaUganda; ^395^ IFEVA/Cátedra de Producción VegetalDepartamento de Producción VegetalFacultad de AgronomíaUniversidad de Buenos Aires/CONICET.Buenos AiresArgentina; ^396^ Directora del Programa Conservación de Biodiversidad en Bosques SubtropicalesCátedra de Desarrollo Sustentable y BiodiversidadFacultad de Ciencias AgrariasUniversidad Nacional de JujuyCIT‐Jujuy CONICET, Fundaciòn CEBioSan Salvador de Jujuy, Argentina; ^397^ Departament de Ciències AmbientalsUniversitat de GironaGironaSpain; ^398^ EntomologyCornell UniversityIthacaNYUSA; ^399^ BotanySchool of Natural SciencesTrinity College DublinDublin 2Ireland; ^400^ Center for Environmental Sciences and Engineering & Department of Ecology and Evolutionary BiologyUniversity of ConnecticutStorrsCTUSA; ^401^ MARETEC, Instituto Superior TécnicoUniversidade de LisboaLisbonPortugal; ^402^ CREA‐ABP, Consiglio per la ricerca in agricoltura e l'analisi dell'economia agraria, Centro di ricerca per l'agrobiologia e la pedologiaFirenzeItaly; ^403^ Ecosystem Management, School of Environment and Rural ScienceUniversity of New EnglandArmidaleNSWAustralia; ^404^ Escuela de BiologíaUniversidad Industrial de SantanderBucaramangaColombia; ^405^ National Center for Ecological Analysis and SynthesisUniversity of California, Santa BarbaraSanta BarbaraCAUSA; ^406^ Department of BioscienceAarhus UniversityAarhus CDenmark; ^407^ The Royal Society for the Protection of Birds (RSPB)Edinburgh ParkEdinburghUK; ^408^ Center for Conservation and Sustainable DevelopmentMissouri Botanical GardenSaint LouisMOUSA; ^409^ Departamento de BiologiaUniversidade Federal de SergipeSão Cristóvão/SeBrazil; ^410^ Life Sciences DepartmentUniversity of AlcalaAlcalá de HenaresSpain; ^411^ Entomology Colletion, Systematics and Biogeography LaboratorySchool of BiologyIndustrial University of SantanderBucaramangaColombia; ^412^ Percy FitzPatrick Institute of African OrnithologyDST/NRF Centre of ExcellenceUniversity of Cape TownRondeboschCape TownSouth Africa; ^413^ School of Animal, Plant and Environmental SciencesUniversity of the WitwatersrandWitsSouth Africa; ^414^ Centro de Ciências Biológicas e da SaúdeUniversidade Federal de Mato Grosso do SulCampo GrandeBrazil; ^415^ Department of Biological SciencesBrock UniversitySt. CatharinesONCanada; ^416^ EdinburghUK; ^417^ Luquillo LTER, Institute for Tropical Ecosystem Studies, College of Natural SciencesUniversity of Puerto Rico at Rio PiedrasSan JuanPRUSA; ^418^ Escuela Nacional de Estudios SuperioresUniversidad Nacional Autónoma de MéxicoMoreliaMexico; ^419^ Science and Conservation DivisionDepartment of Parks and WildlifeManjimupWAAustralia; ^420^ PROPLAME‐PRHIDEB‐CONICETDepartamento de Biodiversidad y Biología ExperimentalFacultad de Ciencias Exactas y NaturalesUniversidad de Buenos Aires, Ciudad Universitaria(CP1428EHA) Ciudad Autónoma de Buenos AiresArgentina; ^421^ ECT Oekotoxikologie GmbHFlörsheim am MainGermany; ^422^ LOEWE Biodiversity and Climate Research Centre BiK‐FFrankfurt/MainGermany; ^423^ Facultad de Ciencias AmbientalesUniversidad de Ciencias Aplicadas y Ambientales U.D.C.ABogotáColombia; ^424^ Catedras CONACYTCIIDIR, Unidad Oaxaca, IPNSanta Cruz Xoxocotlán, Mexico; ^425^ Universidad de Ciencias Aplicadas y Ambientales U.D.C.A.BogotáColombia; ^426^ School of Natural Resources and EnvironmentUniversity of MichiganAnn ArborMIUSA; ^427^ Department of Environmental SciencesUniversity of VirginiaCharlottesvilleVAUSA; ^428^ Blandy Experimental FarmBoyceVAUSA; ^429^ Département des sciences biologiques (SB)Universitédu Québec à Montréal (UQÀM)MontréalQCCanada; ^430^ Facultad de CienciasUniversidad de ChileSantiagoChile; ^431^ School of Geography, Earth and Environmental SciencesUniversity of BirminghamBirminghamUK; ^432^ Institute of Silviculture and Forest ProtectionUniversity of West HungarySopronHungary; ^433^ Red de Ecología FuncionalInstituto de Ecología A.C. Carretera antigua a CoatepecEl Haya, XalapaMexico; ^434^ Biology Centre CASInstitute of EntomologyCeske BudejoviceCzech Republic; ^435^ Faculty of ScienceUniversity of South BohemiaCeske BudejoviceCzech Republic; ^436^ Bishop's UniversitySherbrookeQCCanada; ^437^ CSIRODutton ParkQldAustralia; ^438^ Naturalis Biodiversity CenterCR LeidenThe Netherlands; ^439^ Institute for Tropical Biology and ConservationUniversiti Malaysia Sabah, Jalan UMSKota KinabaluMalaysia; ^440^ Biocentre Klein Flottbek & Botanical GardenUniversity of HamburgHamburgGermany; ^441^ Center for Development Research (ZEF)University of BonnBonnGermany; ^442^ Chair for Landscape ManagementUniversity of FreiburgFreiburgGermany; ^443^ AgResearch LimitedLincoln Research CentreChristchurchNew Zealand; ^444^ Institute for Ecology, Evolution and DiversityGoethe University FrankfurtFrankfurt am MainGermany; ^445^ Biology and Biomedical Sciences DivisionUniversity of BrightonBrightonUK; ^446^ Charles Darwin UniversityBrinkinNTAustralia; ^447^ Lawrence UniversityAppletonWIUSA; ^448^ School of Natural Resources and ExtensionUniversity of Alaska FairbanksFairbanksAKUSA; ^449^ Center for Ecology, Development and ResearchDehradunIndia; ^450^ School of Life SciencesUniversity of KwaZulu‐NatalDurbanSouth Africa; ^451^ Department of Ecology and Natural Resource Management (INA)Norwegian University of Life Sciences (NMBU)ÅsNorway; ^452^ Museum of Natural Science and Department of Biological SciencesLouisiana State UniversityBaton RougeLAUSA; ^453^ Baton RougeLAUSA; ^454^ Department of Life SciencesBen‐Gurion University of the NegevBe'er ShevaIsrael; ^455^ The Yerucham Center of Ornithology and EcologyYeruchamIsrael; ^456^ Instituto de Ciências BiológicasUniversidade Federal do ParáBelémBrazil; ^457^ Organic Research CentreElm FarmNewburyUK; ^458^ United States Department of AgricultureSouth San FranciscoCAUSA; ^459^ Universidad Nacional de ColombiaSede MedellinMedellinColombia; ^460^ Department of Biological SciencesNational University of SingaporeSingaporeSingapore; ^461^ Ecología de Comunidades Ãridas y Semiaridas (EComAS)Departamento de RecursosFacultad de Ciencias Exactas y NaturalesUNLPam.Santa rosaLa PampaUruguay; ^462^ Gobierno Autónomo Departamental Santa CruzSanta Cruz de la SierraBolivia; ^463^ Université du Québec à RimouskiCentre for Northern Research, Centre for Forest StudiesRimouskiQCCanada; ^464^ School of Environmental StudiesUniversity of VictoriaVictoriaBCCanada; ^465^ Museu de Ciències Naturals de GranollersGranollersBarcelonaSpain; ^466^ School of Renewable Natural ResourcesLouisiana State University Agricultural CenterBaton RougeLAUSA; ^467^ Biological Dynamics of Forest Fragments ProjectInstituto Nacional de Pesquisas da AmazôniaManausBrazil; ^468^ Department of Natural Resources and Environmental ManagementUniversity of HawaiiManoaHonoluluHIUSA; ^469^ Key Laboratory of Zoological Systematics and EvolutionInstitute of ZoologyChinese Academy of SciencesChaoyang DistrictBeijingChina; ^470^ State Key Laboratory of Urban and Regional EcologyResearch Center for Eco‐Environmental SciencesChinese Academy of SciencesHaidian DistrictBeijingChina; ^471^ Institute of ZoologyUniversity of Natural Resources and Life SciencesViennaAustria; ^472^ Department of Environmental Science and PolicyDrake UniversityDes MoinesIAUSA; ^473^ Department of BiologyHong Kong Baptist UniversityKowloon Tong, Hong Kong SARChina; ^474^ Zoological DivisionResearch Center For BiologyThe Indonesian Institute of SciencesCibinongBogorIndonesia; ^475^ Section for Ecoinformatics & BiodiversityDepartment of BioscienceAarhus UniversityAarhus CDenmark; ^476^ Department of Zoology, Institute of Ecology and Earth SciencesUniversity of TartuTartuEstonia; ^477^ School of Ecosystem and Forest Science, Faculty of ScienceThe University of MelbourneRichmondVic.Australia; ^478^ Department of BiologySaint Louis UniversitySt. LouisMOUSA; ^479^ MTA‐DE Biodiversity and Ecosystem Services Research GroupDebrecenHungary; ^480^ Insect Ecology GroupDepartment of ZoologyUniversity of CambridgeCambridgeUK; ^481^ Centre for Integrative Ecology, School of Biological SciencesUniversity of CanterburyChristchurchNew Zealand; ^482^ Instituto Neotropical: Pesquisa e ConservaçãoCuritibaBrazil; ^483^ Department of Ecology and TerritorySchool of Environmental and Rural StudiesPontificia Universidad JaverianaBogotaColombia; ^484^ Naturhistorisches Museum BaselLeiter BiowissenschaftenBaselSwitzerland; ^485^ NERC Centre for Ecology & Hydrology, Bush EstatePenicuikEdinburghUK; ^486^ Instituto de BiologiaUniversidade Federal de Uberlândia (UFU)UberlândiaBrazil; ^487^ Institute of Biodiversity and Ecosystem ResearchBulgarian Academy of ScienceSofiaBulgaria; ^488^ Division Forest, Nature, and LandscapeDepartment of Earth & Environmental SciencesKU LeuvenLeuvenBelgium; ^489^ Museu Nacional de História Natural e da CiênciaBorboletário – Depart. ZoologiaLisboaPortugal; ^490^ Departamento de Ciencias Químico‐BiológicasUniversidad de las Américas PueblaCholulaMexico; ^491^ Departamento de Gestión AgrariaUniversidad de Santiago de ChileSantiagoChile; ^492^ Den HaagThe Netherlands; ^493^ Royal Museum for Central Africa – Joint Experimental Molecular UnitTervurenBelgium; ^494^ Vietnam National Museum of NatureVietnam Academy of Science and TechnologyCau GiayHanoiVietnam; ^495^ Botany DepartmentUniversity of OtagoDunedinNew Zealand; ^496^ School for Resource and Environmental StudiesFaculty of ManagementDalhousie UniversityHalifaxNSCanada; ^497^ Key Laboratory of Protection and Development Utilization of Tropical Crop Germplasm Resource, Ministry of Education, College of Horticulture and Landscape AgricultureHainan UniversityHaikouChina; ^498^ College of Life SciencesZhejiang UniversityHangzhouChina; ^499^ Department of BiologyJohn Carroll UniversityUniversity HeightsOHUSA; ^500^ The Environment Institute and School of Earth and Environmental SciencesThe University of AdelaideAdelaideSAAustralia; ^501^ Environmental Futures Research InstituteGriffith UniversityBrisbaneQldAustralia; ^502^ Department of Environmental and Natural ResourcesPresbyterian University CollegeAkropong AkuapemGhana; ^503^ School of Natural Sciences and PsychologyLiverpool John Moores UniversityLiverpoolUK; ^504^ Center for Environmental Sciences & EngineeringUniversity of ConnecticutStorrsCTUSA; ^505^ Department of Ecology & Evolutionary BiologyUniversity of ConnecticutStorrsCTUSA; ^506^ Institute for Biodiversity and Ecosystem Dynamics (IBED)University of AmsterdamGE AmsterdamThe Netherlands; ^507^ NERC Centre for Ecology & HydrologyCrowmarsh GiffordWallingfordUK; ^508^ Institute of Biodiversity Science, School of Life SciencesFudan UniversityShanghaiChina; ^509^ International Institute of Tropical ForestryUSDA Forest Service, Sabana Field Research StationLuquilloPRUSA; ^510^ Tsukuba UniversityIbarakiJapan; ^511^ School of Biological SciencesUniversity of East AngliaNorwich Research ParkNorwichUK; ^512^ State Key Laboratory of Genetic Resources and Evolution, Kunming Institute of ZoologyChinese Academy of SciencesKunmingChina; ^513^ A. N. Severtsov Institute of Ecology and EvolutionMoscowRussia; ^514^ Integrated Environmental Consultants Namibia (IECN)WindhoekNamibia; ^515^ Guangdong Entomological Institute/South China Institute of Endangered AnimalsGuangzhouChina; ^516^ Computational Ecology and Environmental ScienceMicrosoft ResearchCambridgeUK

**Keywords:** data sharing, global biodiversity modeling, global change, habitat destruction, land use

## Abstract

The PREDICTS project—Projecting Responses of Ecological Diversity In Changing Terrestrial Systems (www.predicts.org.uk)—has collated from published studies a large, reasonably representative database of comparable samples of biodiversity from multiple sites that differ in the nature or intensity of human impacts relating to land use. We have used this evidence base to develop global and regional statistical models of how local biodiversity responds to these measures. We describe and make freely available this 2016 release of the database, containing more than 3.2 million records sampled at over 26,000 locations and representing over 47,000 species. We outline how the database can help in answering a range of questions in ecology and conservation biology. To our knowledge, this is the largest and most geographically and taxonomically representative database of spatial comparisons of biodiversity that has been collated to date; it will be useful to researchers and international efforts wishing to model and understand the global status of biodiversity.

## 1 Introduction

Many indicators are available for tracking the state of biodiversity through time, for example, in order to assess progress toward goals such as the Convention on Biological Diversity's 2010 target or the newer Aichi Biodiversity Targets (Pereira et al., 2013; Tittensor et al., 2014). Most of the available indicators are taxonomically or ecologically narrow in scope, and many are based on the global status of species (e.g., Butchart et al., 2010; Tittensor et al., 2014), because of the finality of extinction. However, using a more representative set of taxa and considering local biodiversity offers several advantages. First, average responses of species to human impacts typically vary among higher taxa and ecological guilds (Lawton et al., 1998; McKinney, 1997; Newbold et al., 2014; WWF International, 2014), meaning that indicators need to be broadly based and as representative as possible, if they are to be used as proxies for biodiversity as a whole. Second, the taxa for which most data on trends are available (typically, charismatic groups such as birds or butterflies) are not always the most important for the continued functioning of ecosystems and delivery of ecosystem services (Norris, 2012). Third, although many of the ultimate drivers behind biodiversity loss are global, the most important pressure mechanisms usually act much more locally (Brook, Ellis, Perring, Mackay, & Blomqvist, 2013). Fourth, most ecosystem services and their underpinning processes are mediated by local rather than global biodiversity (Cardinale et al., 2012; Grime, 1998): It is local rather than global functional diversity, for example, that determines how ecosystems function in a given set of conditions (Steffen et al., 2015). Finally, presence/absence and especially abundance of species at a site respond more rapidly to disturbance than extent of geographic distribution or global/national extinction risk (Balmford, Green, & Jenkins, 2003; Collen et al., 2009; Hull, Darroch, & Erwin, 2015), so local changes are likely to be detected before large global changes or extinction.

For these reasons, there is a need to model the response of local biodiversity to human pressures and, thus, to estimate biodiversity changes at local scales, but across a wide spatial domain (ideally globally) and for a wide range of taxa. We therefore need comparable high‐quality data on local biodiversity at different levels of human pressure, from many different taxa and regions. At present, spatial comparisons of how biodiversity responds to variation in pressures provide the only feasible way to collate a large, globally representative evidence base and to model responses to human impacts. Although large temporal datasets are available (e.g., Butchart et al., 2004; Collen et al., 2009; Dornelas et al., 2014; Vellend et al., 2013), they may not be sufficiently representative of anthropogenic pressures for the trends they show to be taken at face value (Gonzalez et al., 2016). Furthermore, in the absence of contemporaneous site‐specific information about pressures, it is not straightforward to use these data to model how biodiversity responds to pressures or to project changes into the future (but see Visconti et al., 2015). Spatially extensive field data of suitable quality and resolution are time‐consuming and expensive to collect. The most convenient and readily available source of suitable biodiversity data is the published literature: Thousands of published papers are based on datasets that would be of value to global modeling efforts. However, it has been rare for such papers to publish data in full, even as supporting information, meaning that many potentially valuable datasets are “dark data” (Hampton et al., 2013), effectively at risk of being lost to science if they have not been lost already.

Since 2012, the PREDICTS project has been collating data on local biodiversity at different levels of human pressure from published papers, where necessary contacting those papers’ corresponding authors to request the underlying biodiversity data, species’ identities, and precise sampling locations. We have enhanced the collated data by scoring site characteristics relating to human pressures such as the predominant land use and how intensively the land is used by humans. We also used the geographical coordinates of the sites to match them to a number of published spatially explicit datasets. The database has already been used to conduct global (e.g., Newbold et al., 2015; Newbold, Hudson, Arnell, et al., 2016), regional (De Palma et al., 2016) and national (Echeverría‐Londoño et al., 2016) analyses of the responses of local biodiversity to land use and related human pressures. The database was first described by Hudson et al. (2014) who published an interim version (March 2014) of the site‐level metadata along with a detailed description of how the database has been collated and validated. Since that time, the database has nearly doubled in size. Here, we describe the status of the database and make available the full species‐level data themselves (not just the site metadata previously released) to facilitate other research, especially into human impacts on ecological assemblages. We also include suggestions for how the database can be used.

## 2 Methods

We sought datasets describing the abundance or occurrence of species, or the diversity of ecological assemblages of species at multiple sites in different land uses or at different levels of other human pressures (e.g., differing levels of land‐use intensity). Data were primarily collated through subprojects on particular regions, land uses, or taxa. We also made general requests for data at conferences and through published articles (Hudson, Newbold, et al., 2013; Hudson et al., 2014; Newbold et al., 2012). Through the course of the project, searches were increasingly targeted toward under‐ or unrepresented regions, biomes, or taxa, in order to mitigate biased coverage in the literature.

To be included in the database, data were required to meet the following criteria: (1) the dataset was part of a published work, or the sampling methods were published; (2) the same sampling procedure was carried out at each site within each study (sampling effort was permitted to vary so long as it was recorded for each site); and (3) we could acquire the geographical coordinates of each sampled site. Where the author of the original publication was unable to supply the geographical coordinates, sites were georeferenced from maps in the publication (Hudson et al., 2014). Sites’ land use—primary vegetation, secondary vegetation (divided according to stage of recovery into mature, intermediate and young; or indeterminate where information on stage was unavailable), plantation forest, cropland, pasture and urban—and, within each land‐use class, intensity—minimal, light and intense—were classified from the description given in the source publication or information subsequently provided by data contributors (see Hudson et al., 2014 for full details). These land‐use categories were chosen to be as compatible as possible with those used in the harmonized land‐use scenarios for 1500–2100 (Hurtt et al., 2011) in order to facilitate spatial and temporal projections of modeled land‐use effects on biodiversity (e.g., Newbold et al., 2015). For some sites, land use and/or use intensity could not be established, so were given missing values.

The data were arranged in a hierarchical structure. The data from an individual published work, typically a published paper, constituted a “DataSource.” Where different sampling methods were used within a DataSource, for example, because different taxonomic groups were collected, and the data were made available separately, the data were divided into separate “Studies.” Data from a given DataSource were also split into multiple Studies if they covered large geographic areas (e.g., several countries), to reduce the effect of biogeographic differences within Studies. Each Study contained a set of sampled “Sites” and “Taxa”; at each Site a set of “Measurements” (typically the abundance or occurrence of a set of taxa) were taken. The provided database extracts contain, for each Site, the raw measurement values, the sampling efforts and, where relevant, the effort‐corrected abundance values (corrected across Sites within a Study by dividing the abundance measurement by sampling effort, assuming that sampled abundances increase linearly with sampling effort, after first rescaling effort values within each Study to a maximum value of one). The measurements were not corrected for different detectability (Hayward et al., 2015; MacKenzie et al., 2002).

It is important to note that the data in the database are often not exactly the same as those used in the source papers. Numbers of sites may differ because datasets provided may have been partial or included extra sites, or because we have aggregated or disaggregated data differently. Likewise, numbers of taxa may differ because of curation or because more data were provided than had been used in the source paper. Because our focus was to make these data as useful as possible for PREDICTS analyses, rather than to act as a repository for datasets from previous publications, it will often not be possible to use these data to replicate the analyses presented in the source papers.

We were limited by the rate at which we could process new data because so many datasets were contributed. This led to the development of a backlog, which we had to clear by the end of the first phase of funding for PREDICTS. During this stage of the project, in order to process all the datasets in hand within the time available, we focused our efforts on the fields shown to be most important in our models to that point (De Palma et al., 2015; Newbold et al., 2014, 2015). As a result, DataSources processed since early 2015 often lack data for some fields, including coordinate precision and maximum linear extent; details of the potentially affected fields are listed in Supporting Information.

Team members were trained in how to score datasets received, using written definitions and descriptions of fields and terms, as well as practice datasets. All data underwent basic validation checks to ensure values entered in each field were appropriate (Hudson et al., 2014). Geographical coordinates were visually inspected on a map after entry into the database, and our software automatically detected coordinates falling outside of the expected country (e.g., because latitude and longitude values were accidentally swapped). For the calculation of biodiversity metrics such as species richness, we accepted the identifications of species provided by the authors of the source publications; these were determined at the time of the original research, and so will not reflect subsequent taxonomic changes or re‐identifications. We also matched taxonomic names to the Catalogue of Life 2013 checklist (COL; Roskov et al., 2013), allowing us to validate many of the names, assess taxonomic coverage and relate measurements to species‐level datasets such as those describing ecological traits. We make available both the original species classifications and those from COL (field names are given in Supporting Information). We reviewed and corrected a number of potential error cases, such as names without a matching COL record, and names for which the higher taxonomic rank of the matching COL record was unexpected (e.g., a COL record for a true fly within a Study that examined birds). Many more validation checks were applied; a complete description is in Hudson et al. (2014).

## 3 Results

### 3.1 Geographical coverage

**Figure 1:**
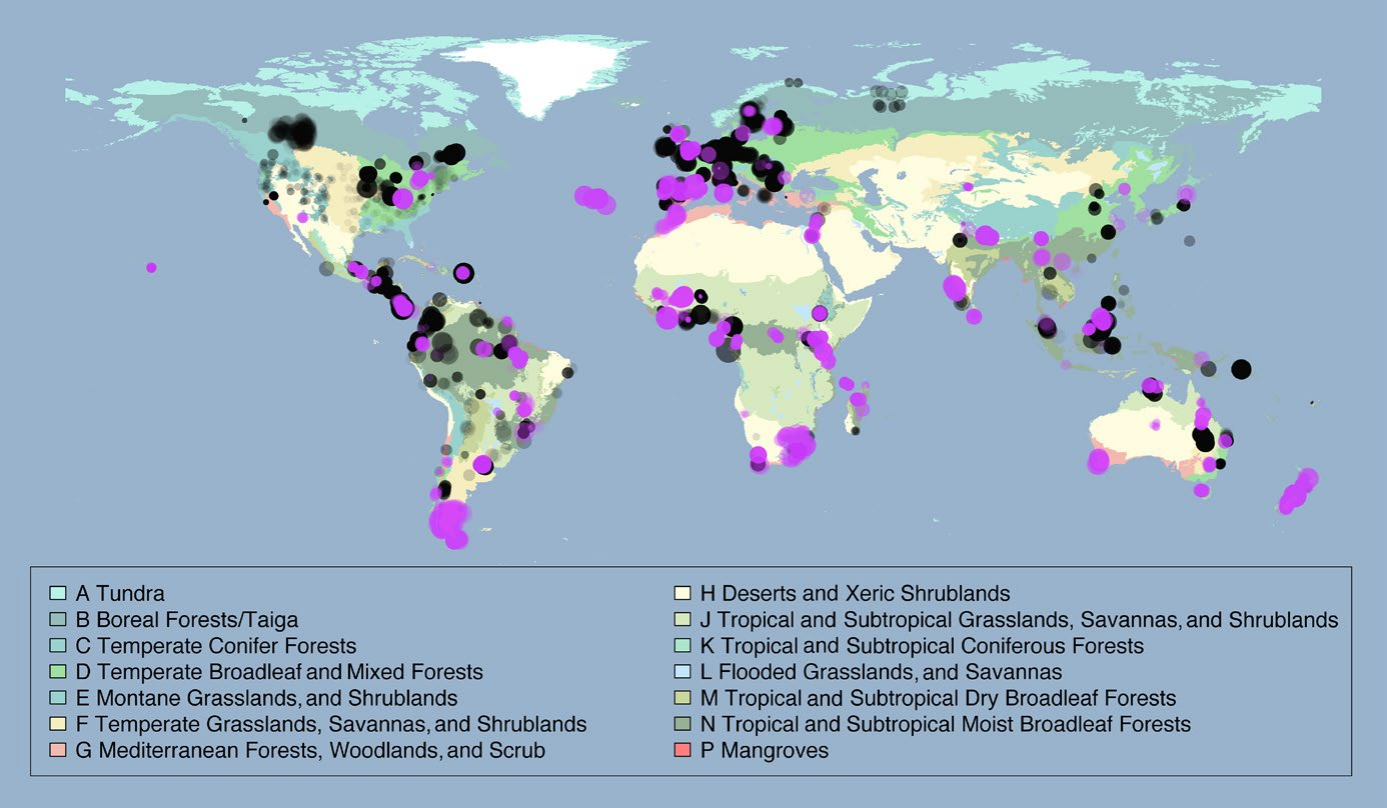
Sampling locations. Map colors indicate biomes, taken from the Terrestrial Ecoregions of the World dataset (The Nature Conservancy, 2009), shown in a geographic (WGS84) projection. Circle radii are proportional to log_10_ of the number of samples at that Site. All circles have the same degree of partial transparency. Sites added to the database since Hudson et al. (2014) are shown in pink

**Figure 2:**
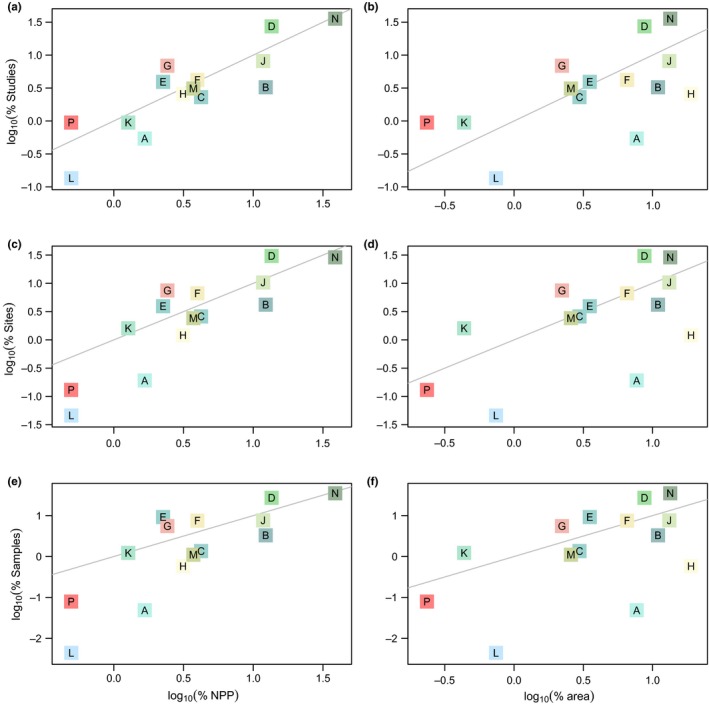
Coverage of biomes. The percentage of Studies (a and b), Sites (c and d), and samples (e and f) against percentages of terrestrial NPP (Net Primary Productivity, computed as in Hudson et al., 2014; a, c, and e) and terrestrial area (b, d, and f). Biome codes and colors are as in Figure 1

**Figure 3:**
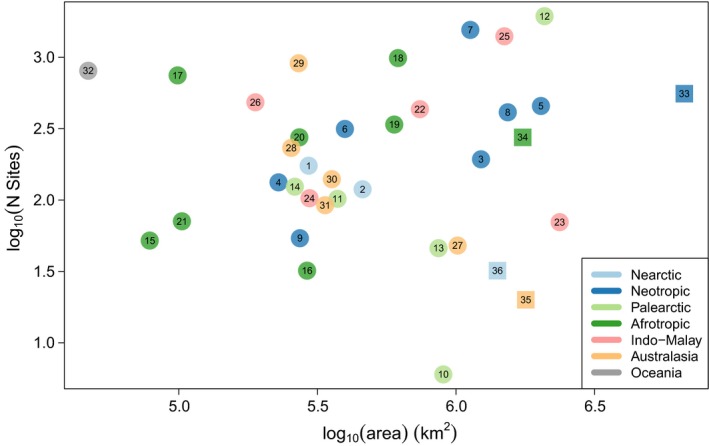
Numbers of Sites against the areas of biodiversity hotspots and of high biodiversity wilderness areas (HBWAs). Hotspots are shown by circles and HBWAs by squares; symbols are colored by the predominant biogeographic realm in which they fall. Hotspots are 1 California Floristic Province, 2 Madrean Pine‐Oak Woodlands, 3 Atlantic Forest, 4 Caribbean Islands, 5 Cerrado, 6 Chilean Winter Rainfall and Valdivian Forests, 7 Mesoamerica, 8 Tropical Andes, 9 Tumbes‐Choco‐Magdalena, 10 Irano‐Anatolian, 11 Japan, 12 Mediterranean Basin, 13 Mountains of Central Asia, 14 Mountains of Southwest China, 15 Cape Floristic Region, 16 Coastal Forests of Eastern Africa, 17 Eastern Afromontane, 18 Guinean Forests of West Africa, 19 Madagascar and the Indian Ocean Islands, 20 Maputaland‐Pondoland‐Albany, 21 Succulent Karoo, 22 Himalaya, 23 Indo‐Burma, 24 Philippines, 25 Sundaland, 26 Western Ghats and Sri Lanka, 27 East Melanesian Islands, 28 Forests of East Australia, 29 New Zealand, 30 Southwest Australia, 31 Wallacea, 32 Polynesia‐Micronesia and HBWAs are 33 Amazonia, 34 Congo Forests, 35 New Guinea, 36 North American Deserts. Unrepresented are the hotspots Caucasus, Horn of Africa, New Caledonia and the HBWA Miombo‐Mopane Woodlands and Savannas

This release of the PREDICTS database contains 3,250,404 records, from 26,114 sampled Sites (Figure 1), collated from 480 DataSources and 666 Studies. The data represent all of the world's 14 terrestrial biomes, in approximate proportion to their contribution to global total primary productivity (Figure 2). The sampled Sites span 94 of the world's countries (including all 17 megadiverse countries; Mittermeier, Gil, & Mittermeier, 1997), 281 of the 814 terrestrial ecoregions (The Nature Conservancy 2009) and 32 of Conservation International's 35 biodiversity hotspots (Myers, Mittermeier, Mittermeier, da Fonseca, & Kent, 2000; circles on Figure 3). Although the database focuses on land use, it also includes data from regions that have so far seen relatively little land‐use change, such as some high biodiversity wilderness areas (Mittermeier et al., 2003; squares on Figure 3).

### 3.2 Taxonomic coverage

**Figure 4:**
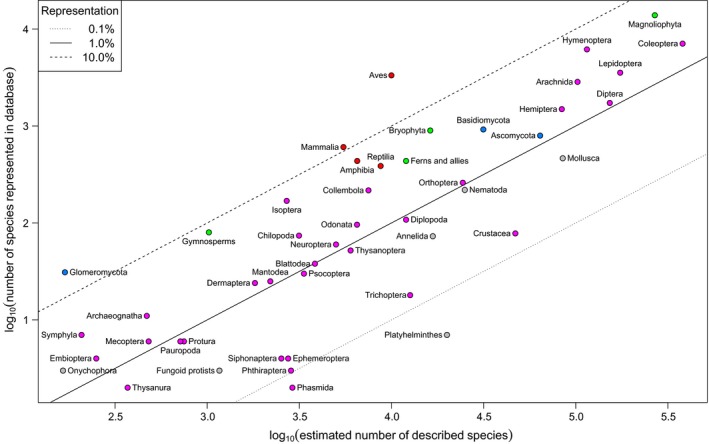
Taxonomic coverage. The numbers of species in our database against the numbers of described species within each of 59 higher taxa, as estimated by Chapman (2009), on logarithmic axes. Vertebrates are shown in red, arthropods in pink, other animals in gray, plants in green, and fungi in blue. The dashed, solid, and dotted lines indicate 10, 1, and 0.1% representation, respectively. Groups with just a single species represented (Diplura and Zoraptera) are not shown

**Figure 5:**
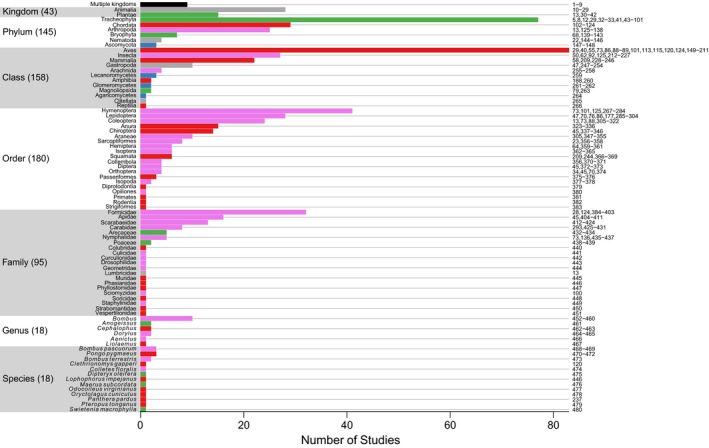
Number of Studies by lowest common taxonomic group. Bars show the number of Studies within each lowest common taxon (so, one Study examined the species *Swietenia macrophylla*, three Studies examined the species *Bombus pascuorum*, ten Studies examined multiple species within the genus *Bombus*, and so on). Colors are as in Figure 4. Numbers on the right are the primary references from which data were taken: 1 Basset et al. (2008), 2 Buscardo et al. (2008), 3 Christensen and Heilmann‐Clausen (2009), 4 Domínguez, Bahamonde, and Muñoz‐Escobar (2012), 5 López‐Quintero, Straatsma, Franco‐Molano, and Boekhout (2012), 6 Nöske et al. (2008), 7 Norton, Espie, Murray, and Murray (2006), 8 Peri, Lencinas, Martínez Pastur, Wardell‐Johnson, and Lasagno (2013), 9 Robinson and Williams (2011), 10 Barratt et al. (2005), 11 Bonham, Mesibov, and Bashford (2002), 12 Boutin, Martin, and Baril (2009), 13 Carpenter et al. (2012), 14 Gaigher and Samways (2010), 15 Ge et al. (2012), 16 Hayward (2009), 17 Leighton‐Goodall, Brown, Hammond, and Eggleton (2012), 18 Muchane et al. (2012), 19 Ngai et al. (2008), 20 Richardson, Richardson, and Soto‐Adames (2005), 21 Schon, Mackay, Minor, Yeates, and Hedley (2008), 22 Schon, Mackay, Yeates, and Minor (2010), 23 Schon, Mackay, and Minor (2011), 24 Smith (2006), 25 Smith, Potts, Woodcock, and Eggleton (2008), 26 Smith, Potts, and Eggleton (2008), 27 Todd et al. (2011), 28 Vasconcelos et al. (2009), 29 Walker, Wilson, Norbury, Monks, and Tanentzap (2014), 30 Baeten, Velghe, et al. (2010), 31 Bakayoko, Martin, Chatelain, Traore, and Gautier (2011), 32 Center for International Forestry Research (CIFOR) (2013a), 33 Center for International Forestry Research (CIFOR) (2013b), 34 Dumont et al. (2009), 35 Firincioglu, Seefeldt, Sahin, and Vural (2009), 36 Haarmeyer, Schmiedel, Dengler, and Bosing (2010), 37 Joubert, Esler, and Privett (2009), 38 Norfolk, Eichhorn, and Gilbert (2013), 39 Page, Qureshi, Rawat, and Kushalappa (2010), 40 Proença, Pereira, Guilherme, and Vicente (2010), 41 Sheil et al. (2002), 42 Wang, Lencinas, Ross Friedman, Wang, and Qiu (2011), 43 Alignier and Deconchat (2013), 44 Baeten, Hermy, Van Daele, and Verheyen (2010), 45 Barlow, Gardner, et al. (2007), 46 Barrico et al. (2012), 47 Baur et al. (2006), 48 Berry et al. (2010), 49 Boutin, Baril, and Martin (2008), 50 Bouyer et al. (2007), 51 Brearley (2011), 52 Brunet et al. (2011), 53 Calviño‐Cancela, Rubido‐Bará, and van Etten (2012), 54 Castro, Lehsten, Lavorel, and Freitas (2010), 55 de Lima, Dallimer, Atkinson, and Barlow (2013), 56 Devineau, Fournier, and Nignan (2009), 57 Fensham, Dwyer, Eyre, Fairfax, and Wang (2012), 58 Fernandez and Simonetti (2013), 59 Fredriksson, Danielsen, and Swenson (2007), 60 Gendreau‐Berthiaume, Kneeshaw, and Harvey (2012), 61 Golodets, Kigel, and Sternberg (2010), 62 Grass, Berens, Peter, and Farwig (2013), 63 Gutierrez et al. (2009), 64 Helden and Leather (2004), 65 Hernández, Delgado, Meier, and Duran (2012), 66 Hietz (2005), 67 Higuera and Wolf (2010), 68 Hylander and Nemomissa (2009), 69 Ishida, Hattori, and Takeda (2005), 70 Kati, Zografou, Tzirkalli, Chitos, and Willemse (2012), 71 Katovai, Burley, and Mayfield (2012), 72 Kessler et al. (2005), 73 Kessler et al. (2009), 74 Kolb and Diekmann (2004), 75 Krauss, Klein, Steffan‐Dewenter, and Tscharntke (2004), 76 Krauss et al. (2010), 77 Kumar and Shahabuddin (2005), 78 Letcher and Chazdon (2009), 79 Louhaichi, Salkini, and Petersen (2009), 80 Lucas‐Borja et al. (2011), 81 Måren (2011), 82 Måren, Bhattarai, and Chaudhary (2013), 83 Marin‐Spiotta, Ostertag, and Silver (2007), 84 Mayfield, Ackerly, and Daily (2006), 85 McNamara, Erskine, Lamb, Chantalangsy, and Boyle (2012), 86 Milder et al. (2010), 87 O'Connor (2005), 88 Paritsis and Aizen (2008), 89 Phalan, Onial, Balmford, and Green (2011), 90 Pincheira‐Ulbrich, Rau, and Smith‐Ramirez (2012), 91 Poggio, Chaneton, and Ghersa (2013), 92 Power and Stout (2011), 93 Power, Kelly, and Stout (2012), 94 Ramesh et al. (2010), 95 Romero‐Duque, Jaramillo, and Perez‐Jimenez (2007), 96 Schmitt, Senbeta, Denich, Preisinger, and Boehmer (2010), 97 Shannon et al. (2008), 98 Siebert (2011), 99 Vassilev, Pedashenko, Nikolov, Apostolova, and Dengler (2011), 100 Williams, Sheahan, and Gormally (2009), 101 Yamaura et al. (2012), 102 Alcala, Alcala, and Dolino (2004), 103 Bicknell and Peres (2010), 104 Centro Agronómico Tropical de Investigación y Enseñanza (CATIE) (2010); Deheuvels, Avelino, Somarriba, and Malézieux (2012), Deheuvels et al. (2014); Rousseau, Deheuvels, Rodriguez Arias, and Somarriba (2012), 105 Craig et al. (2009), 106 Craig et al. (2012), 107 Craig, Grigg, Hobbs, and Hardy (2014), 108 Craig, Stokes, StJ. Hardy, and Hobbs (2015), 109 de Thoisy et al. (2010), 110 Endo et al. (2010), 111 Garden, McAlpine, and Possingham (2010), 112 Kurz, Nowakowski, Tingley, Donnelly, and Wilcove (2014), 113 Kutt and Woinarski (2007), 114 Kutt, Vanderduys, and O'Reagain (2012), 115 Lehouck et al. (2009), 116 Macip‐Ríos and Muñoz‐Alonso (2008), 117 McCarthy, McCarthy, Fuller, and McCarthy (2010), 118 Parry, Barlow, and Peres (2009), 119 Peres and Nascimento (2006), 120 St‐Laurent, Ferron, Hins, and Gagnon (2007), 121 Sung, Karraker, and Hau (2012), 122 Urbina‐Cardona, Olivares‐Perez, and Reynoso (2006), 123 Woinarski and Ash (2002), 124 Woinarski et al. (2009), 125 Billeter et al. (2008); Le Féon et al. (2010), 126 Borges et al. (2006), 127 Cabra‐García, Bermúdez‐Rivas, Osorio, and Chacón (2012), 128 Hanley (2011), 129 Lachat et al. (2006), 130 Cardoso et al. (2009); Meijer, Whittaker, and Borges (2011), 131 Nakamura, Proctor, and Catterall (2003), 132 Norfolk, Abdel‐Dayem, and Gilbert (2012), 133 Poveda, Martinez, Kersch‐Becker, Bonilla, and Tscharntke (2012), 134 Rousseau, Fonte, Tellez, van der Hoek, and Lavelle (2013), 135 Turner and Foster (2009), 136 Uehara‐Prado et al. (2009), 137 Waite (2012); Waite, Closs, Van Heezik, Berry, and Dickinson (2012), 138 Woodcock et al. (2007), 139 Albertos, Lara, Garilleti, and Mazimpaka (2005), 140 Draper, Lara, Albertos, Garilleti, and Mazimpaka (2006), 141 Giordano et al. (2004), 142 Hylander and Weibull (2012), 143 Medina et al. (2010), 144 Hu and Cao (2008), 145 Wu, Fu, Chen, and Chen (2002), 146 Zhang, Li, and Liang (2010), 147 Giordani et al. (2010), 148 Giordani (2012), 149 Aben, Dorenbosch, Herzog, Smolders, and Van Der Velde (2008), 150 Arbeláez‐Cortés, Rodríguez‐Correa, and Restrepo‐Chica (2011), 151 Aumann (2001), 152 Azhar et al. (2013), 153 Azman et al. (2011), 154 Azpiroz and Blake (2009), 155 Báldi, Batáry, and Erdos (2005), 156 Barlow, Mestre, Gardner, and Peres (2007), 157 Bóçon (2010), 158 Borges (2007), 159 Brandt et al. (2013), 160 Cerezo, Conde, and Poggio (2011), 161 Chapman and Reich (2007), 162 Cockle, Leonard, and Bodrati (2005), 163 Dallimer, Parnell, Bicknell, and Melo (2012), 164 Dawson et al. (2011), 165 Dures and Cumming (2010), 166 Edenius, Mikusinski, and Bergh (2011), 167 Farwig, Sajita, and Boehning‐Gaese (2008), 168 Flaspohler et al. (2010), 169 Gomes, Oostra, Nijman, Cleef, and Kappelle (2008); Oostra, Gomes, and Nijman (2008), 170 Hassan et al. (2013), 171 Ims and Henden (2012), 172 Lantschner, Rusch, and Peyrou (2008), 173 Lasky and Keitt (2010), 174 Latta, Tinoco, Astudillo, and Graham (2011), 175 Mallari et al. (2011), 176 Doulton, Marsh, Newman, Bird, and Bell (2007), 177 Marsh, Lewis, Said, and Ewers (2010), 178 Miranda, Politi, and Rivera (2010), 179 Moreno‐Mateos et al. (2011), 180 Munyekenye, Mwangi, and Gichuki (2008), 181 Naidoo (2004), 182 Naithani and Bhatt (2012), 183 Naoe, Sakai, and Masaki (2012), 184 Ndang'ang'a, Njoroge, and Githiru (2013), 185 Neuschulz, Botzat, and Farwig (2011), 186 O'Dea and Whittaker (2007), 187 Owiunji and Plumptre (1998), 188 Pearman (2002), 189 Politi, Hunter Jr. and Rivera (2012), 190 Pons and Wendenburg (2005), 191 Ranganathan, Chan, and Daily (2007), 192 Ranganathan, Daniels, Chandran, Ehrlich, and Daily (2008), 193 Reid, Harris, and Zahawi (2012), 194 Rey‐Benayas, Galvan, and Carrascal (2010), 195 Reynolds and Symes (2013), 196 Rosselli (2011), 197 Sam, Koane, Jeppy, and Novotny (2014), 198 Santana, Porto, Gordinho, Reino, and Beja (2012), 199 Shahabuddin and Kumar (2006, 2007), 200 Sheldon, Styring, and Hosner (2010), 201 Sodhi et al. (2010), 202 Soh, Sodhi, and Lim (2006), 203 Sosa, Benz, Galea, and Poggio Herrero (2010), 204 Stouffer, Johnson, Bierregaard, Richard, and Lovejoy (2011), 205 Suarez‐Rubio and Thomlinson (2009), 206 Vergara and Simonetti (2004), 207 Verhulst, Báldi, and Kleijn (2004), 208 Waite, Closs, van Heezik, and Dickinson (2013), 209 Wang, Bao, Yu, Xu, and Ding (2010), 210 Wunderle, Henriques, and Willig (2006), 211 Li, Zou, Zhang, and Sheldon (2013), 212 Bates et al. (2011), 213 Blake, Westbury, Woodcock, Sutton, and Potts (2011), 214 Blanche, Ludwig, and Cunningham (2006), 215 Cleary et al. (2004), 216 Farwig et al. (2009), 217 Franzén and Nilsson (2008), 218 Kohler, Verhulst, van Klink, and Kleijn (2008), 219 Litchwark (2013), 220 Meyer, Gaebele, and Steffan‐Dewenter (2007), 221 Jauker, Krauss, Jauker, and Steffan‐Dewenter (2013); Meyer, Jauker, and Steffan‐Dewenter (2009), 222 Mudri‐Stojnic, Andric, Jozan, and Vujic (2012), 223 Quintero, Morales, and Aizen (2010), 224 Rader, Bartomeus, Tylianakis, and Laliberte (2014), 225 Schüepp, Herrmann, Herzog, and Schmidt‐Entling (2011), 226 Summerville (2011), 227 Vergara and Badano (2009), 228 Bernard, Fjeldsa, and Mohamed (2009), 229 Cáceres, Nápoli, Casella, and Hannibal (2010), 230 Cassano, Barlow, and Pardini (2014), 231 Danquah, Oppong, and Nutsuakor (2012), 232 Garmendia, Arroyo‐Rodriguez, Estrada, Naranjo, and Stoner (2013), 233 Gheler‐Costa, Vettorazzi, Pardini, and Verdade (2012), 234 Granjon and Duplantier (2011), 235 Henschel (2008), 236 Hoffmann and Zeller (2005), 237 Kittle, Watson, Chanaka Kumara, and Nimalka Sanjeewani (2012), 238 Lantschner, Rusch, and Hayes (2012), 239 Martin, Gheler‐Costa, Lopes, Rosalino, and Verdade (2012), 240 McShea et al. (2009), 241 Mena and Medellín (2010), 242 Nakagawa, Miguchi, and Nakashizuka (2006), 243 O'Farrell, Donaldson, Hoffman, and Mader (2008), 244 Scott et al. (2006), 245 Sridhar, Raman, and Mudappa (2008), 246 Wells, Kalko, Lakim, and Pfeiffer (2007), 247 Hylander, Nilsson, and Gothner (2004), 248 Kappes, Katzschner, and Nowak (2012), 249 Oke and Chokor (2009), 250 Oke (2013), 251 Schilthuizen, Liew, Bin Elahan, and Lackman‐Ancrenaz (2005), 252 Ström, Hylander, and Dynesius (2009), 253 Torre, Bros, and Santos (2014), 254 Wronski et al. (2014), 255 Freire and Motta (2011), 256 Lo‐Man‐Hung, Gardner, Ribeiro‐Júnior, Barlow, and Bonaldo (2008), 257 Shochat, Stefanov, Whitehouse, and Faeth (2004), 258 Zaitsev, Chauvat, Pug, and Wolters (2002), 259 Walker, Crittenden, Young, and Prystina (2006), 260 Malonza and Veith (2012), 261 Alguacil, Torrecillas, Hernandez, and Roldan (2012), 262 Brito, Goss, de Carvalho, Chatagnier, and van Tuinen (2012), 263 Baral and Katzensteiner (2009), 264 Robles, Carmaran, and Lopez (2011), 265 Römbke, Schmidt, and Höfer (2009), 266 Luja, Herrando‐Perez, Gonzalez‐Solis, and Luiselli (2008), 267 Cameron et al. (2011), 268 Cunningham, Schellhorn, Marcora, and Batley (2013), 269 Fowler (2014), 270 Gould et al. (2013), 271 Lentini, Martin, Gibbons, Fischer, and Cunningham (2012), 272 Malone et al. (2010), 273 Marshall, West, and Kleijn (2006), 274 Oertli, Muller, and Dorn (2005), 275 Osgathorpe, Park, and Goulson (2012), 276 Quaranta et al. (2004), 277 Richards et al. (2011), 278 Samnegård, Persson, and Smith (2011), 279 Schüepp, Rittiner, and Entling (2012), 280 Shuler, Roulston, and Farris (2005), 281 Smith‐Pardo and Gonzalez (2007), 282 Tonietto, Fant, Ascher, Ellis, and Larkin (2011), 283 Tylianakis, Klein, and Tscharntke (2005), 284 Verboven, Brys, and Hermy (2012), 285 Barlow, Overal, Araujo, Gardner, and Peres (2007), 286 Berg, Ahrné, Öckinger, Svensson, and Söderström (2011), 287 Bobo, Waltert, Fermon, Njokagbor, and Muhlenberg (2006), 288 Cleary and Mooers (2006), 289 D'Aniello, Stanislao, Bonelli, and Balletto (2011), 290 de Sassi, Lewis, and Tylianakis (2012), 291 Dolia, Devy, Aravind, and Kumar (2008), 292 Hawes et al. (2009), 293 Ishitani, Kotze, and Niemela (2003), 294 Krauss, Steffan‐Dewenter, and Tscharntke (2003), 295 Littlewood (2008), 296 Pe'er, Maanen, Turbe, Matsinos, and Kark (2011), 297 Safian, Csontos, and Winkler (2011), 298 Summerville and Crist (2002), 299 Summerville, Conoan, and Steichen (2006), 300 Sutrisno (2010), 301 Uehara‐Prado, Brown, Spalding, and Lucci Freitas (2007), 302 Verdasca et al. (2012), 303 Vu (2005), 304 Vu (2009), 305 Banks, Sandvik, and Keesecker (2007), 306 Barratt et al. (2012), 307 Blanche and Cunningham (2005), 308 Buse, Levanony, Timm, Dayan, and Assmann (2008), 309 Elek and Lovei (2007), 310 Ewers, Thorpe, and Didham (2007), 311 Gaublomme, Hendrickx, Dhuyvetter, and Desender (2008), 312 Gray, Slade, Mann, and Lewis (2014), 313 Jonsell (2012), 314 Légaré, Hébert, and Ruel (2011), 315 Mico, Garcia‐Lopez, Brustel, Padilla, and Galante (2013), 316 Noreika (2009), 317 Numa, Verdu, Rueda, and Galante (2012), 318 Nyeko (2009), 319 Otavo, Parrado‐Rosselli, and Noriega (2013), 320 Rodrigues, Uchoa, and Ide (2013), 321 Sugiura, Tsuru, Yamaura, and Makihara (2009), 322 Verdú et al. (2007), 323 Adum, Eichhorn, Oduro, Ofori‐Boateng, and Rodel (2013), 324 de Souza, de Souza, and Morato (2008), 325 Eigenbrod, Hecnar, and Fahrig (2008), 326 Faruk, Belabut, Ahmad, Knell, and Garner (2013), 327 Furlani, Ficetola, Colombo, Ugurlucan, and De Bernardi (2009), 328 Gutierrez‐Lamus (2004), 329 Hilje and Aide (2012), 330 Isaacs‐Cubides and Urbina‐Cardona (2011), 331 Ofori‐Boateng et al. (2013), 332 Pethiyagoda and Manamendra‐Arachchi (2012), 333 Pillsbury and Miller (2008), 334 Pineda and Halffter (2004), 335 Vallan (2002), 336 Watling, Gerow, and Donnelly (2009), 337 Castro‐Luna, Sosa, and Castillo‐Campos (2007), 338 Clarke, Rostant, and Racey (2005), 339 Fukuda, Tisen, Momose, and Sakai (2009), 340 MacSwiney, Vilchis, Clarke, and Racey (2007), 341 Presley, Willig, Wunderle, Joseph, and Saldanha (2008), 342 Sedlock et al. (2008), 343 Shafie, Sah, Latip, Azman, and Khairuddin (2011), 344 Struebig, Kingston, Zubaid, Mohd‐Adnan, and Rossiter (2008), 345 Threlfall, Law, and Banks (2012), 346Willig et al. (2007), 347 Alcayaga, Pizarro‐Araya, Alfaro, and Cepeda‐Pizarro (2013), 348 Buddle and Shorthouse (2008), 349 Clark, Gerard, and Mellsop (2004), 350 Kapoor (2008), 351 Lo‐Man‐Hung et al. (2011), 352 Magura, Horvath, and Tothmeresz (2010), 353 Malumbres‐Olarte et al. (2014), 354 Paradis and Work (2011), 355 Raub, Hoefer, Scheuermann, and Brandl (2014), 356 Alberta Biodiversity Monitoring Institute (ABMI) (2013), 357 Arroyo, Iturrondobeitia, Rad, and Gonzalez‐Carcedo (2005), 358 Zaitsev, Wolters, Waldhardt, and Dauber (2006), 359 Kőrösi, Batáry, Orosz, Rédei, and Báldi (2012), 360 Littlewood, Pakeman, and Pozsgai (2012), 361 Moir, Brennan, Koch, Majer, and Fletcher (2005), 362 Carrijo, Brandao, de Oliveira, Costa, and Santos (2009), 363 Oliveira, Carrijo, and Brandão (2013), 364 Reis and Cancello (2007), 365 Zeidler, Hanrahan, and Scholes (2002), 366 D'Cruze and Kumar (2011), 367 Fabricius, Burger, and Hockey (2003), 368 Pelegrin and Bucher (2012), 369 Urbina‐Cardona, Londoño‐Murcia, and García‐Ávila (2008), 370 Chauvat, Wolters, and Dauber (2007), 371 Fiera (2008), 372 Savage, Wheeler, Moores, and Taillefer (2011), 373 Virgilio, Backeljau, Emeleme, Juakali, and De Meyer (2011), 374 Andersen, Ludwig, Lowe, and Rentz (2001), 375 Otto and Roloff (2012), 376 Zimmerman, Bell, Woodcock, Palmer, and Paloniemi (2011), 377 Hornung, Tothmeresz, Magura, and Vilisics (2007), 378 Magrini, Freitas, and Uehara‐Prado (2011), 379 Laurance and Laurance (1996), 380 Bragagnolo, Nogueira, Pinto‐da‐Rocha, and Pardini (2007), 381 Herrera, Wright, Lauterbur, Ratovonjanahary, and Taylor (2011), 382 Jung and Powell (2011), 383 Bartolommei, Mortelliti, Pezzo, and Puglisi (2013), 384 Andersen and Hoffmann (2011), 385 Armbrecht, Perfecto, and Silverman (2006), 386 Bihn, Verhaagh, Braendle, and Brandl (2008), 387 Buczkowski (2010), 388 Buczkowski and Richmond (2012), 389 Delabie et al. (2009), 390 Dominguez‐Haydar and Armbrecht (2010), 391 Fayle et al. (2010), 392 Floren, Freking, Biehl, and Linsenmair (2001), 393 Frizzo and Vasconcelos (2013), 394 Gove, Majer, and Rico‐Gray (2005), 395 Gunawardene, Majer, and Edirisinghe (2010), 396 Hashim, Akmal, Jusoh, and Nasir (2010), 397 Kone, Konate, Yeo, Kouassi, and Linsenmair (2010), 398 Maeto and Sato (2004), 399 Roth, Perfecto, and Rathcke (1994), 400 Schmidt, Fraser, Carlyle, and Bassett (2012), 401 Uehara‐Prado (2005), 402 Vasconcelos (1999), 403 Vasconcelos, Vilhena, and Caliri (2000), 404 Fierro, Cruz‐Lopez, Sanchez, Villanueva‐Gutierrez, and Vandame (2012), 405 Hanley (2005), 406 Julier and Roulston (2009), 407 Liow, Sodhi, and Elmqvist (2001), 408 Nielsen et al. (2011), 409 Parra‐H and Nates‐Parra (2007), 410 Rasmussen (2009), 411 Winfree, Griswold, and Kremen (2007), 412 da Silva (2011), 413 Davis and Philips (2005), 414 Filgueiras, Iannuzzi, and Leal (2011), 415 Gardner, Hernandez, Barlow, and Peres (2008), 416 Horgan (2009), 417 Jacobs, Scholtz, Escobar, and Davis (2010), 418 Navarrete and Halffter (2008), 419 Navarro, Roman, Gomez, and Perez (2011), 420 Noriega, Realpe, and Fagua (2007), 421 Noriega, Palacio, Monroy‐G, and Valencia (2012), 422 Rös, Escobar, and Halffter (2012), 423 Silva, Costa, Moura, and Farias (2010), 424 Slade, Mann, and Lewis (2011), 425 Gu, Zhen‐Rong, and Dun‐Xiao (2004), 426 Koivula, Hyyrylainen, and Soininen (2004), 427 Liu, Axmacher, Wang, Li, and Yu (2012), 428 Noreika and Kotze (2012), 429 Rey‐Velasco and Miranda‐Esquivel (2012), 430 Vanbergen, Woodcock, Watt, and Niemela (2005), 431 Weller and Ganzhorn (2004), 432 Aguilar‐Barquero and Jiménez‐Hernández (2009), 433 Carvalho, Ferreira, Lima, and de Carvalho (2010), 434 Svenning (1998), 435 Benedick et al. (2006), 436 Fermon, Waltert, Vane‐Wright, and Muhlenberg (2005), 437 Ribeiro and Freitas (2012), 438 Breedt, Dreber, and Kellner (2013), 439 Scott, Setterfield, Douglas, and Andersen (2010), 440 Cagle (2008), 441 Johnson, Gómez, and Pinedo‐Vasquez (2008), 442 Su, Zhang, and Qiu (2011), 443 Gottschalk, De Toni, Valente, and Hofmann (2007), 444 Axmacher et al. (2009), 445 García, Ortiz Zapata, Aguayo, and D'Elia (2013), 446 Jolli and Pandit (2011), 447 Saldaña‐Vázquez, Sosa, Hernández‐Montero, and López‐Barrera (2010), 448 Nicolas, Barriere, Tapiero, and Colyn (2009), 449 Sakchoowong, Nomura, Ogata, and Chanpaisaeng (2008), 450 García‐R, Cárdenas‐H, and Castro‐H (2007), 451 Yoshikura, Yasui, and Kamijo (2011), 452 Connop, Hill, Steer, and Shaw (2011), 453 Darvill, Knight, and Goulson (2004), 454 Diekötter, Walther‐Hellwig, Conradi, Suter, and Frankl (2006), 455 Goulson, Lye, and Darvill (2008), 456 Goulson et al. (2010), 457 Hanley et al. (2011), 458 Hatfield and LeBuhn (2007), 459 McFrederick and LeBuhn (2006), 460 Redpath, Osgathorpe, Park, and Goulson (2010), 461 Schumann, Wittig, Thiombiano, Becker, and Hahn (2011), 462 Nakashima, Inoue, and Akomo‐Okoue (2013), 463 Wiafe and Amfo‐Otu (2012), 464 Peters, Fischer, Schaab, and Kraemer (2009), 465 Peters, Lung, Schaab, and Waegele (2011), 466 Matsumoto, Itioka, Yamane, and Momose (2009), 467 Rubio and Simonetti (2011), 468 Herrmann, Westphal, Moritz, and Steffan‐Dewenter (2007), 469 Knight et al. (2009), 470 Ancrenaz, Goossens, Gimenez, Sawang, and Lackman‐Ancrenaz (2004), 471 Felton, Engstrom, Felton, and Knott (2003), 472 Knop, Ward, and Wich (2004), 473 Ewers, Bartlam, and Didham (2013), 474 Davis, Murray, Fitzpatrick, Brown, and Paxton (2010), 475 Hanson, Brunsfeld, Finegan, and Waits (2008), 476 Strauch and Eby (2012), 477 Ramos‐Robles, Gallina, and Mandujano (2013), 478 Ferreira and Alves (2005, 2009), 479 Luskin (2010), 480 Grogan et al. (2008)

Records in the PREDICTS database represent 47,044 species (see Hudson et al., 2014 for how species numbers are estimated in the face of imprecise taxon names), which is over 2% of the number thought to have been formally described (Chapman, 2009)—29,737 animals, 15,545 plants, 1,759 fungi, and three protists. The taxonomic distribution of taxa in the database is in rough proportion to the numbers of described species in major taxonomic groups of animals and plants (Figure 4), and the data represent more than 1% as many species as have been described in the following groups: Amphibia, Arachnida, Archaeognatha, Ascomycota, Aves, Basidiomycota, Bryophyta, Chilopoda, Coleoptera, Collembola, Dermaptera, Diptera, Embioptera, Ferns and allies, Glomeromycota, Gymnosperms, Hemiptera, Hymenoptera, Isoptera, Lepidoptera, Magnoliophyta, Mammalia, Mantodea, Mecoptera, Neuroptera, Odonata, Onychophora, Orthoptera, Reptilia, Symphyla and Zoraptera (Figure 4). Vertebrates—and especially birds—are overrepresented owing to biases in the published literature (Figure 4), but less so than in many other data compilations (e.g., over half of the records currently in the Global Biodiversity Information Facility [GBIF] are of birds; www.gbif.org, accessed in April 2016). Most Studies in the PREDICTS database sampled at least multiple families, if not multiple orders, classes, phyla, or even kingdoms (Figure 5). However, some Studies sampled only a single family, genus, or even species (Figure 5).

### 3.3 Temporal coverage

**Figure 6:**
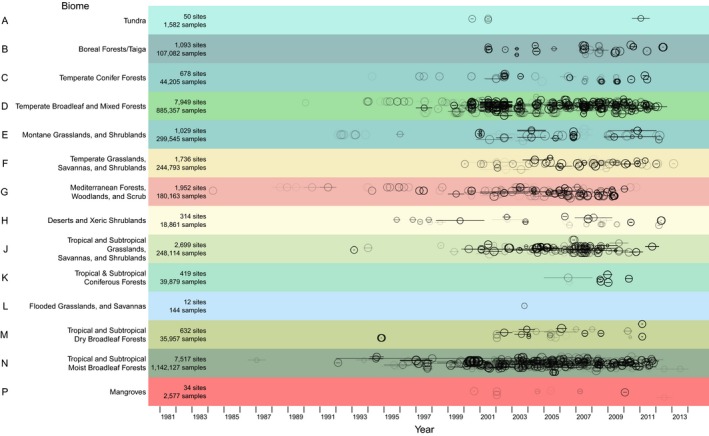
Spatiotemporal sampling coverage. Site sampling dates are shown by biome. Each Site is represented by a circle and line. Circle radii are proportional to log_10_ of the number of samples at that Site. Circle centers are at the midpoints of Site sampling dates; lines indicate the start and end dates of sampling. *Y*‐values have been jittered at the Study level. Circles and lines have the same degree of partial transparency. Biome colors and letters are as in Figure 1

We focused primarily on data sampled since 2000 because most global layers describing human pressure are collected after this year and, in particular, to facilitate use of contemporaneous Moderate‐resolution Imaging Spectroradiometer (MODIS) remotely sensed data (Justice et al., 1998; Tuck et al., 2014) in modeling. However, in filling certain taxonomic and geographic gaps, we also collated some data that were sampled before 2000 (Figure 6). Data are sparse after 2012 because of the natural time lags between data collection in the field, publication and then assimilation into the PREDICTS database (Figure 6).

### 3.4 Data access and structure

This 2016 release of the database—the complete dataset and also site‐level summaries—is available on the data portal of the Natural History Museum, London (doi: 10.5519/0066354) as comma‐separated variable (CSV) files and as RDS files, the latter for use with the R statistical modeling language (R Core Team 2015; RDS files were generated using R 3.3.1). A complete description of the columns in the extracts, along with a visualization of the database schema, is given in Supporting Information. This paper makes all the data in this version of the database freely available to anyone wishing to use them for any purpose. The terms of the license require that anyone publishing research based on these data should cite this paper and/or the original sources of the data used, as appropriate. The dataset at doi: 10.5519/0066354 contains bibliographic information for all DataSources in both CSV and BibTeX formats.

## 4 Discussion

The PREDICTS database is designed to be able to address a range of questions about how land use and related pressures have influenced the occurrence and abundance of species and the diversity of ecological assemblages. The highly structured nature of the data, with comparable surveys having been carried out at each Site within a Study, was chosen to facilitate such modeling. Table 1 identifies a range of long‐standing general questions for which the PREDICTS data may be useful, referencing early papers addressing questions of each type. It also outlines the steps required to tackle each kind of question, in conjunction with other information about the Sites and species where necessary, and refers to papers that have performed so.

**Table 1:** Questions that could be answered using the PREDICTS database

Question		Early example references	Approach	Example using PREDICTS database
*Questions about taxa*				
Q 1.	What factors influence the occurrence and/or abundance of a particular focal species?	Austin, Nicholls, and Margules (1990)	Filter to remove species not of interest. Merge PREDICTS data with data on any additional site‐level characteristics of interest. One possible analytical approach is to model effects of site characteristics on presence‐absence and log (abundance when present) separately, the first with binomial errors and the second with Gaussian errors, while accounting for among‐Study differences (e.g., using mixed‐effects models).	–
Q 2.	Do changes in land‐use facilitate success of invasive species?	Dukes and Mooney (1999), Theoharides and Dukes (2007)	Obtain lists of invasive species for the regions of interest and model presence‐absence and/or abundance of invasives as above.	–
Q 3.	Which ecological attributes of species make them more or less sensitive to human pressures?	McKinney (1997), Davies, Margules, and Lawrence (2000), Cardillo et al. (2005)	Merge PREDICTS data with species‐level data on traits of interest. Model how site and species characteristics affect presence‐absence and log (abundance when present) separately as above, accounting for Study‐level and taxon‐level differences (e.g., using mixed‐effects models).	Newbold et al. (2014), De Palma et al. (2015)
Q 4.	Which taxa have species that are more sensitive to human pressures, and which have less sensitive species?	Lawton et al. (1998), Mace and Balmford (2000), Gibson et al. (2011)	Add taxonomic group into models above as a fixed effect interacting with other fixed effects.	–
Q 5.	Are phylogenetically distinct species particularly sensitive?	Gaston and Blackburn (1997), Purvis, Agapow, Gittleman, and Mace (2000)	Analyze phylogenetic distinctiveness or unique evolutionary history in the same way as ecological attributes.	–
Q 6.	What are the relationships between geographic range size or occupancy and abundance?	Brown (1984)	Merge PREDICTS data with species‐level data on range sizes or occupancy. Filter to the land uses of interest (e.g., primary vegetation if the focus is on natural systems), and examine within‐Study relationship between abundance and relative range size or occupancy.	–
Q 7.	Do suitability estimates from environmental niche models predict abundance?	VanDerWal, Shoo, Johnson, and Williams (2009)	Use other data on occurrences of species to fit niche models for all species in within selected Studies and thereby estimate suitability of each Site. Various modeling options are then possible depending on the precise question: for example, fit land use interacting with suitability when modeling abundance in order to test whether any correlation depends on land use.	–
*Questions about sites*				
Q 8.	Which land uses and other Site‐level pressures have the strongest net impact on levels of local biodiversity?	Lawton et al. (1998), Gibson et al. (2011)	Aggregate biodiversity data within a site to estimate relevant diversity metric (e.g., within‐sample species richness, total abundance, rarefaction‐based richness, species evenness). Merge Site‐level biodiversity data with any additional data on Site‐level characteristics of interest (e.g., from remotely sensed data) if required. Model Site‐level diversity as a function of Site characteristics while accounting for among‐Study differences (e.g., using mixed‐effects models).	fig 1b,c in Newbold et al. (2015)
Q 9.	How do land use and other pressures reduce compositional intactness?	Scholes and Biggs (2005)	Because net changes are affected by gains of non‐native species as well as losses of those originally present, modeling compositional intactness gives a more sensitive indication of human impacts. Model Site‐level abundance as a function of pressures as above, and how compositional similarity to assemblages in primary vegetation differs among land uses. Combine these models to estimate the Biodiversity Intactness Index (Scholes & Biggs, 2005)—the average abundance of a diverse set of species, relative to their abundance in an unimpacted assemblage.	Newbold, Hudson, Arnell, et al. (2016)
Q 10.	Do land use and related pressures influence community trait values?	Garnier et al. (2007)	Combine data on species’ occurrences or abundance with trait data to obtain average or community‐weighted mean trait values, which can then be modeled like the Site‐level response variables above.	fig 1d in Newbold et al. (2015)
Q 11.	Does the biotic response to a given pressure vary regionally?	Gibson et al. (2011)	Add region as a fixed effect and test for interaction with other fixed effects.	–
Q 12.	Which characteristics of Sites (e.g., duration of human impact and rate of climate change) mean that given land‐use changes have particularly severe effects on biodiversity?	Balmford (1996),Travis (2003)	Merge Site‐level diversity data with Site‐level data on characteristics to be tested and assess the interaction of these variables with land use.	Gray et al. (2016)
Q 13.	How accurate are global land‐use data?	Giri, Zhu, and Reed (2005)	Use Site‐level land‐use data to calculate the receiver operating characteristic curve (i.e., sensitivity versus false‐positive rate), using the area under the curve to quantify agreement. An extension of this could be to use the PREDICTS Site‐level land use data as input into land use/land cover classification procedures, for example, by the remote sensing community, or at least use PREDICTS data to cross‐check and validate land use and land cover maps with independent PREDICTS data.	Hoskins et al. (2016)
*Questions above the site level*				
Q 14.	Is beta diversity lower in human‐dominated than more natural land uses?	Tylianakis et al. (2005)	Estimate desired measures of similarity among Sites within studies. Model how biotic similarity among Sites depends on similarity of other attributes (including characteristics from remote sensing or Dynamic Global Ecosystem Models if required), accounting for among‐Study differences (e.g., using mixed‐effects models).	Newbold, Hudson, Hill, et al. (2016)
Q 15.	Are land‐sparing or land‐sharing strategies optimal for local biodiversity?	Green, Cornell, Scharlemann, and Balmford (2005)	Analyze species by Sites and by Study and relate back to Q. 1. The overarching question about sparing versus sharing can be addressed by looking at the individual responses of species to land‐use intensity, as measured by yield suggested by Green et al. (2005); this requires data on agricultural yields at relevant Sites in the PREDICTS database.	–
*Other questions*				
Q 16.	How accurate are current extent of occurrence/range maps, for example, those produced by International Union for Conservation of Nature (2016)?	–	Cross‐check existing extents of occurrence and ranges with PREDICTS data.	–
Q 17.	How representative are species catalogues?	–	Query clade‐level (e.g., The Plant List, World List of Mammalian Species, Platnick's Spider Catalogue) and aggregated (e.g., Encyclopedia of Life and Catalogue of Life) lists with the Latin binomials and trinomials that were provided to PREDICTS by the data collectors. Subquestions include How does coverage vary among taxonomic groups? How does coverage depend on region? Are there substantial differences among the aggregated services? How well are synonyms and homonyms represented and resolved?	–

Changes in attitudes to—and the increasing ease of—data sharing have contributed to rapid growth in open compilations of structured biodiversity data and related pressure data targeted toward particular kinds of research question. Examples of data types featured in such compilations include population time series (e.g., Inchausti & Halley, 2001), assemblage time series (e.g., Dornelas et al., 2014), assemblage inventories (e.g., Thibault, Supp, Giffin, White, & Ernest, 2011), and species traits (e.g., Madin et al., 2016). Other projects have collated or are collating large compilations of structured biodiversity data, such as BIOFRAG (Pfeifer et al., 2014; habitat fragmentation), BIOTIME (The BioTIME Research Group, 2016; detailed time‐series data, still being compiled) and GLOBIO3 (Alkemade et al., 2009; pristine versus disturbed habitats, not publicly available).

The largest open compilation of biodiversity data is the Global Biodiversity Information Facility (GBIF; www.gbif.org), which aggregates mostly unstructured species occurrence data. The unstructured nature of most GBIF data limits the range of questions to which they can easily be put, although they are increasingly used in modeling species distributions (e.g., Pineda & Lobo, 2008) and habitat suitability (e.g., Ficetola, Rondinini, Bonardi, Baisero, & Padoa‐Schioppa, 2015). As of April 2016, GBIF holds over 560 million georeferenced occurrence records of around 1.5 million species, although coverage is taxonomically uneven (e.g., most records are of birds) and patchy even among the best‐recorded groups (Meyer, Kreft, Guralnick, & Jetz, 2015).

Databases of species traits continue to be collated and published, and many of them are relevant to taxa in the PREDICTS database. Recent examples include mammalian generation time (Pacifici et al., 2013), a variety of mammalian traits (Jones et al., 2009), foraging attributes of birds and mammals (Wilman et al., 2014), field metabolic rates of birds and mammals (Hudson, Isaac, & Reuman, 2013) and functional traits of vascular plants (Kattge et al., 2011). Additional databases provide more abstract concepts such as species’ threat status (International Union for Conservation of Nature, 2016) and estimates of the degrees of protection required (Convention on International Trade in Endangered Species of Wild Fauna and Flora, 2016). Relating such data with measurements in the PREDICTS database makes possible investigation into how traits mediate species’ responses to changes in land use and land‐use intensity. Examples of published analyses have examined habitat specialization and geographical range size of birds and mammals (Newbold et al., 2014), functional traits of vascular plants (Bernhardt‐Römermann et al., 2011) and a range of morphometric, physiological, and functional traits of bees (De Palma et al., 2015); see Table 1, Q. 3.

Although our targeting of data from underrepresented biomes and taxa (Hudson et al., 2014) reduces the effects of geographic and taxonomic biases in available data, the PREDICTS database nonetheless has many limitations, of which four are particularly important to note. First, our individual datasets seldom take a whole‐ecosystem perspective, being instead taxonomically or ecologically restricted; consequently, our data shed little light on how trophic webs or other interactions are affected by human pressures. Second, even within the groups sampled, our data do not provide complete inventories of the species that would be found with comprehensive sampling; thus, failure to record a species from a Site does not provide strong evidence of absence. Third, Latin binomials were not available for a sizeable fraction of the species in our DataSources, limiting the prospects for linking the observations of occurrence and abundance to other information about the species (e.g., functional traits; Kattge et al., 2011). Last, because our database was designed to test hypotheses about local‐scale variation in biodiversity, it is not particularly informative about large‐scale biodiversity patterns such as the latitudinal gradient in species richness or how pressures with a coarse spatial grain (e.g., atmospheric nitrogen deposition; Simkin et al., 2016) influence Site‐level diversity.

When using the PREDICTS database, or indeed any database, to model biodiversity responses, it is important to be aware of potential mismatches in scale between Site‐level data and pressure data such as MODIS remotely sensed data (Justice et al., 1998) and the harmonized land‐use scenarios (Hurtt et al., 2011) and also between Site‐level response variables and the scales of interest. The PREDICTS database contains some structural features that help with these issues. First, we assigned the Site‐level land use and land‐use intensity classifications based on the authors’ descriptions of the habitats so these classifications do not suffer from the problem of scale mismatch. Second, Sites are represented as precisely as possible: Sites often represent individual quadrats, traps, or other points within a broader sampling regime (such as a transect), and we recorded (as latitude and longitude) the coordinates of each Site rather than aggregating them into coarser summaries across the broader sampling regime. Third, where the relevant information was available, we also recorded the maximum extent of sampling as a linear value in meters (for 22,199 Sites, see Hudson et al. (2014) for details). Users of the database therefore have flexibility in deciding how measurements in the PREDICTS database are related to available pressure data. Possible solutions to scale mismatches between biodiversity data and pressure data would be (1) to exclude from analyses any Sites where the extent of sampling is substantially greater than the grain size of the pressure data or (2) to conduct some sort of spatial averaging of the pressure data. Novel methods have been published both for downscaling pressure data (e.g., Hoskins et al., 2016) and for upscaling local biodiversity measurements to estimate changes in gamma diversity over broader areas (e.g., Azaele et al., 2015); both approaches offer potential solutions to mismatches in scale.

The PREDICTS database continues to increase in size and currently contains a further 22 Studies with embargo dates that prevent their inclusion in this release. We intend to publish occasional updates to make these data freely available. We have also received a number of further offers of datasets that we hope to incorporate into the database and include in future releases. There are three priority categories of data that we are still seeking actively: bees from outside Western Europe; soil invertebrates and fungi; and geographic islands. The current database focuses entirely on spatial “control–impact” comparisons. A follow‐on project that has recently begun focuses instead on temporal comparisons, collating data from “before–after” and (especially) “before–after–control–impact” studies of the effects of land‐use change on terrestrial assemblages. We are therefore seeking datasets, linked to peer‐reviewed publications, of comparable species‐level surveys conducted at each sampling location, with temporal changes in land use and/or land‐use intensity. If corresponding authors of such papers wish to offer their data, please complete our online form, available at www.predicts.org.uk/pages/contribute.html. As with PREDICTS, the new project will seek to make its data freely available.

## Conflict of Interest

None declared.
