# CORRIGENDUM

**DOI:** 10.1002/ece3.10313

**Published:** 2023-07-23

In the recent article by Hudson et al. (2017), the authors would like to update the URL for the PREDICTS project which is included in the Abstract and Discussion sections as follow:

https://www.nhm.ac.uk/our‐science/our‐work/biodiversity/predicts.html

The article has been corrected online.

## References

[ece310313-bib-0001] Hudson, L. N. , Newbold, T. , Contu, S. , Hill, S. L. L. , Lysenko, I. , De Palma, A. , Phillips, H. R. P. , Alhusseini, T. I. , Bedford, F. E. , Bennett, D. J. , Booth, H. , Burton, V. J. , Chng, C. W. T. , Choimes, A. , Correia, D. L. P. , Day, J. , Echeverría‐Londoño, S. , Emerson, S. R. , Gao, D. , & …. Purvis, A. (2017). The database of the PREDICTS (projecting responses of ecological diversity in changing terrestrial systems) project. Ecology and Evolution, 7(1), 145–188. 28070282 10.1002/ece3.2579 PMC5215197

