# Supplementary material for: The database of the PREDICTS (Projecting Responses of Ecological Diversity In Changing Terrestrial Systems) project
Source: Ecol Evol. 2016 Dec 16;7(1):145–88. doi: 10.1002/ece3.2579 (PMC5215197; doi:10.1002/ece3.2579)
Supplement: Supplementary file 1 [file ECE3-7-145-s001.pdf]

# The database of the PREDICTS (Projecting Responses of Ecological Diversity In Changing Terrestrial Systems) Project
# Supporting File S1

Lawrence N Hudson, Tim Newbold *et al.*

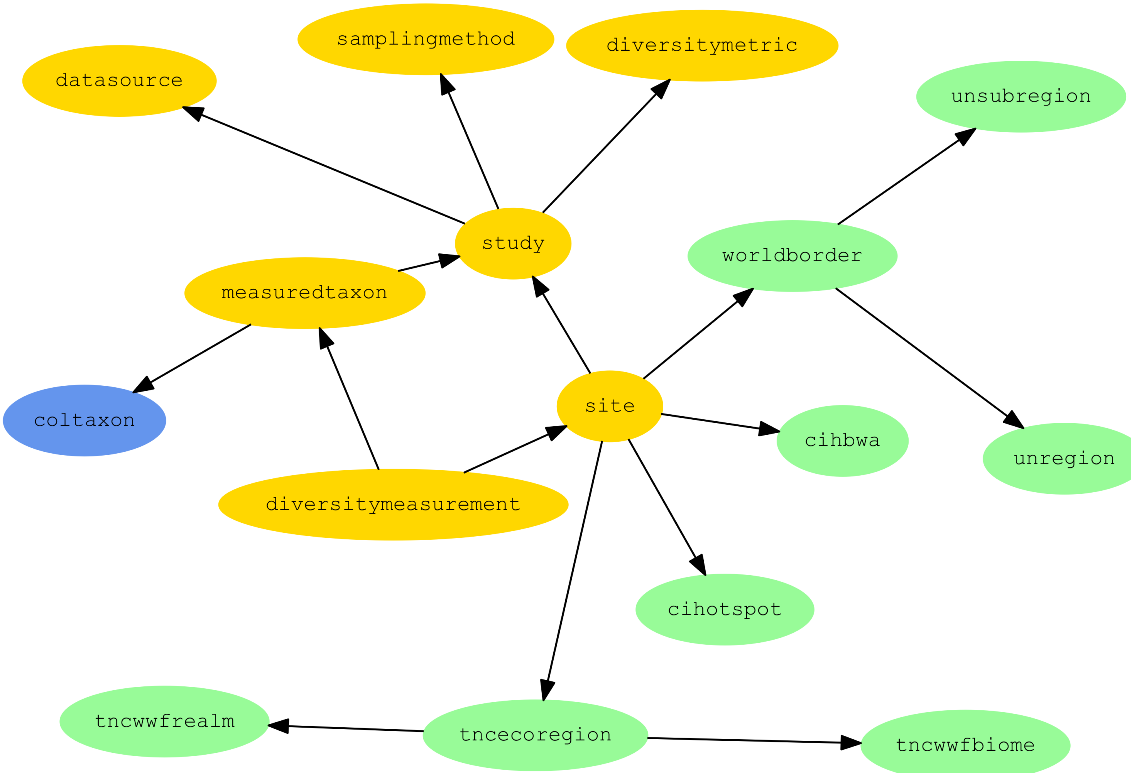


Figure S1: Database schema. Diversity data in yellow, GIS data in green and Catalogue of Life data in blue. The diversity tables `datasource`, `study`, `site`, `measuredtaxon` and `diversitymeasurement` follow the structure described in 'Methods' in the main text and in Hudson et al. (2014): a `datasource` is associated with one or more `study` records, each of which is associated with two or more `sites` records. Each `study` record is associated with a single `samplingmethod`, a single `diversitymetric` and one or more `measuredtaxon` records. A `diversitymeasurement` record exists for each represented combination of `measuredtaxon` and `site`, reflecting the site-by-species matrix structure of the data. Each `worldborder` GIS record is associated with a single `unregion` record and a single `unsubregion` record, representing a country together with its United Nations region and subregion (Thematic Mapping, 2008). Each `tncecoregion` GIS record is associated with a single `tncwwfbiome` record and a single `tncwwfrealm` record, representing an ecoregion together with its biome and realm (The Nature Conservancy, 2009). Each `site` record is associated with a single `worldborder` record and a single `tncecoregion` record. Where coordinates fall within a Conservation International biodiversity hotspot and/or a Conservation International high biodiversity wilderness area the `site` record will be associated with a single `cihotspot` (Myers et al., 2000) record and/or a single `cihbwa` (Mittermeier et al., 2003) record respectively. Each `measuredtaxon` is associated with a single `coltaxon` record, taken from Catalogue of Life 2013 checklist (Roskov et al., 2013).

| Column | Database table | Site extract? | Diversity extract? | Type | Guaranteed to be non empty? | Notes | Validation |
|---|---|---|---|---|---|---|---|
| ***DataSource*** | | | | | | | |
| Source_ID | `datasource` | Yes | Yes | String | Yes | ID for the DataSource. | Unique among DataSources. |
| Reference | | Yes | Yes | String | Yes | Reference for the DataSource in the main text. | |
| ***Study*** | | | | | | | |
| Study_number | `study` | Yes | Yes | Integer | Yes | | For a DataSource with $n$ Studies, $1 <= value <= n$. |
| Study_name | `study` | Yes | Yes | String | Yes | | Unique within Source_ID. |
| SS | | Yes | Yes | String | Yes | Concatenation of Source_ID and Study. | |
| Diversity_metric | `diversitymetric` | Yes | Yes | String | Yes | | |
| Diversity_metric_unit | `diversitymetric` | Yes | Yes | String | Yes | | |
| Diversity_metric_type | `diversitymetric` | Yes | Yes | String | Yes | One of:<br>• Abundance<br>• Occurrence<br>• Species richness | |
| Diversity_metric_is_effort_sensitive | `diversitymetric` | Yes | Yes | Logical | Yes | | |
| Diversity_metric_is_suitable_for_Chao | `diversitymetric` | Yes | Yes | Logical | Yes | | |
| Sampling_method | `samplingmethod` | Yes | Yes | String | Yes | | |
| Sampling_effort_unit | `samplingmethod` | Yes | Yes | String | Yes | | |
| Study_common_taxon | `study` | Yes | Yes | String | No | The lowest taxonomic group (kingdom, phylum, class, order, family, genus or species) that is common to all taxa within this Study. Empty for Studies that examined taxa in multiple kingdoms. | |
| Rank_of_study_common_taxon | `study` | Yes | Yes | String | No | The rank of Study_common_taxon. Empty for Studies that examined taxa in multiple kingdoms. | |
| Site_number | `site` | Yes | Yes | Integer | Yes | | For a Study with $n$ Sites, $1 <= value <= n$. |
| Site_name | `site` | Yes | Yes | String | Yes | Where requested by data providers, the names of some Sites have been replaced with 'Site ' + Site_number. | Unique within Study. |
| Block | `site` | Yes | Yes | Integer | No | Within a Study either:<br>• Empty for all Sites<br>• Non-empty for all Sites and at least two different values among Sites | |

| Column | Database table | Site extract? | Diversity extract? | Type | Guaranteed to be non empty? | Notes | Validation |
|---|---|---|---|---|---|---|---|
| ***Site*** | | | | | | | |
| SSS | | Yes | Yes | String | Yes | Concatenation of Source_ID, Study_number and Site_number. | |
| SSB | | Yes | Yes | String | Yes | Concatenation of Source_ID, Study_number and Block. | |
| SSBS | | Yes | Yes | String | Yes | Concatenation of Source_ID, Study_number, Block and Site_number. | |
| Sample_start_earliest | `site` | Yes | Yes | Date | Yes | | |
| Sample_end_latest | `site` | Yes | Yes | Date | Yes | | Sample_start_earliest<= *value*. |
| Sample_midpoint | `site` | Yes | Yes | Date | Yes | Mid-point of Sample_start_earliest and Sample_end_latest. | |
| Sample_date_resolution | `site` | Yes | Yes | String | Yes | One of:<br>• day<br>• month<br>• year | |
| Max_linear_extent_metres | `site` | Yes | Yes | Number | No | The maximum linear extent of sampling in metres. | If present, $0 < value$. |
| Habitat_patch_area_square_metres | `site` | Yes | Yes | Number | No | Habitat_patch_area expressed in square metres. | |
| Sampling_effort | `site` | Yes | Yes | Number | No | In units given in Sampling_effort_unit. Where sampling effort did not vary among sites within a study, we set the Sampling_effort to 1. | If present, $0 < value$. |
| Rescaled_sampling_effort | `site` | Yes | Yes | Number | No | Sampling effort rescaled to be between 0 and 1 within the Study i.e., Sampling_effort / max(Sampling_effort within this Study). | If present, $0 < value$. |
| Habitat_as_described | `site` | Yes | Yes | String | No | Free text description of habitat. Where requested by data providers, the habitat descriptions of some Sites have been removed from the data extract. | |

| Column | Database table | Site extract? | Diversity extract? | Type | Guaranteed to be non empty? | Notes | Validation |
|---|---|---|---|---|---|---|---|
| ***Site*** | | | | | | | |
| Predominant_land_use | `site` | Yes | Yes | String | Yes | One of:<br>• Primary vegetation<br>• Young secondary vegetation<br>• Intermediate secondary vegetation<br>• Mature secondary vegetation<br>• Secondary vegetation (indeterminate age)<br>• Plantation forest<br>• Pasture<br>• Cropland<br>• Urban<br>• Cannot decide<br>This column was named Predominant_habitat in Hudson et al. (2014). | |
| Source_for_predominant_land_use | `site` | Yes | Yes | String | No | One of:<br>• Direct from publication / author<br>• Google map<br>May be empty for data collated before this information was captured. This column was named Source_for_predominant_habitat in Hudson et al. (2014). | |
| Use_intensity | `site` | Yes | Yes | String | Yes | One of:<br>• Minimal use<br>• Light use<br>• Intense use<br>• Cannot decide | |
| Km_to_nearest_edge_of_habitat | `site` | Yes | Yes | Number | No | Distance in km to the nearest edge of habitat supporting high diversity. A negative value indicates that the Site was within the high-diversity habitat. | |

| Column | Database table | Site extract? | Diversity extract? | Type | Guaranteed to be non empty? | Notes | Validation |
|---|---|---|---|---|---|---|---|
| ***Site*** | | | | | | | |
| Years_since_fragmentation_or_conversion | `site` | Yes | Yes | Number | No | Years since fragmentation or conversion to present land cover (Primary habitat) or since start of recovery (Secondary habitat). | If present, $0 < value < 500$. |
| Transect_details | `site` | Yes | Yes | String | No | Free text. Where requested by data providers, the transect details of some Sites have been removed from the data extract. | |
| Coordinates_method | `site` | Yes | Yes | String | Yes | One of: <br>• Direct from publication / author<br>• Georeferenced | |
| Longitude | `site` | Yes | Yes | Number | No | Where requested by data providers, the coordinates for some Sites have been removed from the data extract. | $-180 <= value <= 180$ |
| Latitude | `site` | Yes | Yes | Number | No | Where requested by data providers, the coordinates for some Sites have been removed from the data extract. | $-90 <= value <= 90$ |
| Country_distance_metres | `site` | Yes | Yes | Integer | Yes | If zero, Site latitude and longitude were within the matching World Borders 0.3 GIS polygon (Thematic Mapping, 2008). If greater than zero, the value is the distance in metres to the nearest WorldBorders GIS polygon. | |
| Country | `worldborder` | Yes | Yes | String | Yes | Coordinates matched to a World Borders GIS polygon. | |
| UN_subregion | `unregion` | Yes | Yes | String | Yes | Coordinates matched to a World Borders GIS polygon. | |
| UN_region | `unsubregion` | Yes | Yes | String | Yes | Coordinates matched to a World Borders GIS polygon. | |

| Column | Database table | Site extract? | Diversity extract? | Type | Guaranteed to be non empty? | Notes | Validation |
|---|---|---|---|---|---|---|---|
| ***Site*** | | | | | | | |
| Ecoregion_distance_metres | `site` | Yes | Yes | Number | Yes | If zero, Site latitude and longitude were within the matching Terrestrial ecoregions of the world GIS polygon (The Nature Conservancy, 2009). If greater than zero, the value is the distance in metres to the nearest Terrestrial ecoregions of the world GIS polygon. | |
| Ecoregion | `tncecoregion` | Yes | Yes | String | Yes | Coordinates matched to a Terrestrial ecoregions of the world GIS polygon. | |
| Biome | `tncwwfbiome` | Yes | Yes | String | Yes | Coordinates matched to a Terrestrial ecoregions of the world GIS polygon. | |
| Realm | `tncwwfrealm` | Yes | Yes | String | Yes | Coordinates matched to a Terrestrial ecoregions of the world GIS polygon. | |
| Hotspot | `cihotspot` | Yes | Yes | String | No | Coordinates matched to a biodiversity hotspots GIS polygon (Myers et al., 2000). Empty if Site did not fall within a hotspot polygon. | |
| Wilderness_area | `cihbwa` | Yes | Yes | String | No | Coordinates matched to a high biodiversity wilderness areas GIS polygon (Mittermeier et al., 2003). Empty if Site did not fall within a wilderness area polygon. | |
| N_samples | | Yes | No | Integer | Yes | The number of samples at this Site. | $0 <= value$ |
| Higher_taxa | | Yes | No | String | No | Comma-separated list of higher taxonomic groups studied at this Site (see Higher_taxon column, below). Empty if either no taxa measured at the Site were sufficiently well resolved for a higher taxonomic group to be computed, or if no taxa were detected at the Site. | |

| Column | Database table | Site extract? | Diversity extract? | Type | Guaranteed to be non empty? | Notes | Validation |
|---|---|---|---|---|---|---|---|
| ***Taxon*** | | | | | | | |
| Taxon_number | `measuredtaxon` | No | Yes | Integer | Yes | | For a Study with $n$ taxa, $1 <= value <= n$. |
| Taxon_name_entered | `measuredtaxon` | No | Yes | String | Yes | Name of the taxon as provided by the data contributor. | Unique within Study. |
| Indication | `measuredtaxon` | No | Yes | String | No | A free-text description of the higher taxonomic group of this taxon. | |
| Parsed_name | `measuredtaxon` | No | Yes | String | Yes | The result of parsing Taxon_name_entered. | |
| Taxon | `coltaxon` | No | Yes | String | Yes | Matching taxon in the Catalogue of Life 2013 checklist (COL; Roskov et al., 2013). | |
| COL_ID | `coltaxon` | No | Yes | Integer | Yes | The ID of Taxon in COL. | |
| Name_status | `coltaxon` | No | Yes | String | Yes | From COL. | |
| Rank | `coltaxon` | No | Yes | String | Yes | From COL. | |
| Kingdom | `coltaxon` | No | Yes | String | Yes | From COL. | |
| Phylum | `coltaxon` | No | Yes | String | Yes | From COL. | |
| Class | `coltaxon` | No | Yes | String | Yes | From COL. | |
| Order | `coltaxon` | No | Yes | String | Yes | From COL. | |
| Family | `coltaxon` | No | Yes | String | Yes | From COL. | |
| Genus | `coltaxon` | No | Yes | String | Yes | From COL. | |
| Species | `coltaxon` | No | Yes | String | Yes | From COL. | |

| Column | Database table | Site extract? | Diversity extract? | Type | Guaranteed to be non empty? | Notes | Validation |
|---|---|---|---|---|---|---|---|
| ***Taxon*** | | | | | | | |
| Best_guess_binomial | | No | Yes | String | No | COL did not recognize all of the Latin binomials that were given to us so we employed the following scheme:<br>• The value of the Species column if Rank contains 'Species'<br>• The first two words of the Species column if Rank contains 'Infraspecies'<br>• The first two words of the Parsed_name column if Rank contains neither 'Infraspecies' nor 'Species' and Parsed_name contains two or more words<br>• Empty in other cases | |
| Higher_taxon | | No | Yes | String | No | The higher-taxonomic group that this taxon belongs to. | |
| ***Measurement*** | | | | | | | |
| Measurement | `diversitymeasurement` | No | Yes | Number | Yes | The biodiversity measurement of the Taxon at the Site in the Study, in units of Diversity_metric_unit. | $0 <= value$ |
| Effort_corrected_measurement | `diversitymeasurement` | No | Yes | Number | Yes | Where Diversity_metric_is_effort_sensitive is TRUE, the biodiversity measurement corrected for sampling effort (i.e., Measurement / Rescaled_sampling_effort). Where Diversity_metric_is_effort_sensitive is FALSE, the same value as Measurement. | $0 <= value$ |

Table S1: Database extract columns. Database extract columns are grouped by the levels of the structure described in 'Methods' in the main text: DataSource, Study, Site, Taxon and Measurement. Database tables are described in Figure S1. Where 'Database table' is blank, values were not stored in the database but were computed from other values. 'Type' is one of 'String' (textual information), 'Logical' (either 'TRUE' or 'FALSE'), 'Date' (in the form 'YYYY-MM-DD'), 'Integer' or 'Number'.

## References


Hudson, L. N., Newbold, T., Contu, S., Hill, S. L. L., Lysenko, I., De Palma, A., Phillips, H. R. P., Senior, R. A., Bennett, D. J., Booth, H., Choimes, A., Correia, D. L. P., Day, J., Echeverría-Londoño, S., Garon, M., Harrison, M. L. K., Ingram, D. J., Jung, M., Kemp, V., Kirkpatrick, L., Martin, C. D., Pan, Y., White, H. J., Aben, J., Abrahamczyk, S., Adum, G. B., Aguilar-Barquero, V., Aizen, M. A., Ancrenaz, M., Arbeláez-Cortés, E., Armbrecht, I., Azhar, B., Azpiroz, A. B., Baeten, L., Báldi, A., Banks, J. E., Barlow, J., Batáry, P., Bates, A. J., Bayne, E. M., Beja, P., Berg, a., Berry, N. J., Bicknell, J. E., Bihn, J. H., Böhning-Gaese, K., Boekhout, T., Boutin, C., Bouyer, J., Brearley, F. Q., Brito, I., Brunet, J., Buczkowski, G., Buscardo, E., Cabra-García, J., Calviño Cancela, M., Cameron, S. A., Cancello, E. M., Carrijo, T. F., Carvalho, A. L., Castro, H., Castro-Luna, A. A., Cerda, R., Cerezo, A., Chauvat, M., Clarke, F. M., Cleary, D. F. R., Connop, S. P., D'Aniello, B., da Silva, P. G., Darvill, B., Dauber, J., Dejean, A., Diekötter, T., Dominguez-Haydar, Y., Dormann, C. F., Dumont, B., Dures, S. G., Dynesius, M., Edenius, L., Elek, Z., Entling, M. H., Farwig, N., Fayle, T. M., Felicioli, A., Felton, A. M., Ficetola, G. F., Filgueiras, B. K. C., Fonte, S. J., Fraser, L. H., Fukuda, D., Furlani, D., Ganzhorn, J. U., Garden, J. G., Gheler-Costa, C., Giordani, P., Giordano, S., Gottschalk, M. S., Goulson, D., Gove, A. D., Grogan, J., Hanley, M. E., Hanson, T., Hashim, N. R., Hawes, J. E., Hébert, C., Helden, A. J., Henden, J.-A., Hernández, L., Herzog, F., Higuera-Diaz, D., Hilje, B., Horgan, F. G., Horváth, R., Hylander, K., Isaacs-Cubides, P., Ishitani, M., Jacobs, C. T., Jaramillo, V. J., Jauker, B., Jonsell, M., Jung, T. S., Kapoor, V., Kati, V., Katovai, E., Kessler, M., Knop, E., Kolb, A., Kőrösi, A., Lachat, T., Lantschner, V., Le Féon, V., LeBuhn, G., Légaré, J.-P., Letcher, S. G., Littlewood, N. A., López-Quintero, C. A., Louhaichi, M., Lövei, G. L., Lucas-Borja, M. E., Luja, V. H., Maeto, K., Magura, T., Mallari, N. A., Marin-Spiotta, E., Marshall, E. J. P., Martínez, E., Mayfield, M. M., Mikusinski, G., Milder, J. C., Miller, J. R., Morales, C. L., Muchane, M. N., Muchane, M., Naidoo, R., Nakamura, A., Naoe, S., Nates-Parra, G., Navarrete Gutierrez, D. A., Neuschulz, E. L., Noreika, N., Norfolk, O., Noriega, J. A., Nöske, N. M., O'Dea, N., Oduro, W., Ofori-Boateng, C., Oke, C. O., Osgathorpe, L. M., Paritsis, J., Parra-H, A., Pelegrin, N., Peres, C. A., Persson, A. S., Petanidou, T., Phalan, B., Philips, T. K., Poveda, K., Power, E. F., Presley, S. J., Proença, V., Quaranta, M., Quintero, C., Redpath-Downing, N. A., Reid, J. L., Reis, Y. T., Ribeiro, D. B., Richardson, B. A., Richardson, M. J., Robles, C. A., Römbke, J., Romero-Duque, L. P., Rosselli, L., Rossiter, S. J., Roulston, T. H., Rousseau, L., Sadler, J. P., Sáfián, S., Saldaña-Vázquez, R. A., Samnegård, U., Schüepp, C., Schweiger, O., Sedlock, J. L., Shahabuddin, G., Sheil, D., Silva, F. A. B., Slade, E. M., Smith-Pardo, A. H., Sodhi, N. S., Somarriba, E. J., Sosa, R. A., Stout, J. C., Struebig, M. J., Sung, Y.-H., Threlfall, C. G., Tonietto, R., Tóthmérész, B., Tscharntke, T., Turner, E. C., Tylianakis, J. M., Vanbergen, A. J., Vassilev, K., Verboven, H. A. F., Vergara, C. H., Vergara, P. M., Verhulst, J., Walker, T. R., Wang, Y., Watling, J. I., Wells, K., Williams, C. D., Willig, M. R., Woinarski, J. C. Z., Wolf, J. H. D., Woodcock, B. A., Yu, D. W., Zaitsev, A. S., Collen, B., Ewers, R. M., Mace, G. M., Purves, D. W., Scharlemann, J. P. W. & Purvis, A. (2014) The PREDICTS database: a global database of how local terrestrial biodiversity responds to human impacts. *Ecology and Evolution*, 4 (24), 4701–4735. 10.1002/ece3.1303.

Mittermeier, R. A., Mittermeier, C. G., Brooks, T. M., Pilgrim, J. D., Konstant, W. R., da Fonseca, G. A. B. & Kormos, C. (2003) Wilderness and biodiversity conservation. 100 (18), 10309–10313. 10.1073/pnas.1732458100.

Myers, N., Mittermeier, R. A., Mittermeier, C. G., da Fonseca, G. A. B. & Kent, J. (2000) Biodiversity hotspots for conservation priorities. *Nature*, 403 (6772), 853–858. 10.1038/35002501.

Roskov, Y., Kunz, T., Paglinawan, L., Orrell, T., Nicolson, D., Culham, A., Bailly, N., Kirk, P., Bourgoin, T., Baillargeon, G., Hernandez, F. & De Wever, A. (2013) Species 2000 & Catalogue of Life, 2013 Annual Checklist. **URL:** *http://catalogueoflife.org/annual-checklist/2013/*

The Nature Conservancy (2009) Terrestrial ecoregions of the world. **URL:** *http://maps.tnc.org/gis_data.html*

Thematic Mapping (2008) World borders. **URL:** *http://thematicmapping.org/downloads/world_borders.php*
